# Microwave‐Assisted Synthesis of Cu/Co‐Based Nanoheterostructures for High‐Efficiency Alcohol Oxidation

**DOI:** 10.1002/advs.202505581

**Published:** 2025-07-25

**Authors:** Xuesong Zhang, Jaume Gázquez, Arturo Pajares, Dino Tonti, Pablo Guardia

**Affiliations:** ^1^ Institut de Ciència de Materials de Barcelona (ICMAB‐CSIC) Campus Universitari Bellaterra 08193 Spain; ^2^ Materials & Chemistry Flemish Institute for Technological Research (VITO NV) Boeretang 200 Mol 2400 Belgium

**Keywords:** alcohol oxidation reaction, ethanol oxidation reaction ‐eor, hybrid water splitting, methanol oxidation reaction ‐mor, nanoheterostructures

## Abstract

Hybrid water splitting, using methanol or ethanol oxidation reactions (MOR and EOR) at the counter electrode during electrochemical hydrogen generation, offers an efficient alternative to the sluggish oxygen evolution reaction (OER). This study reports Cu/Co‐based core‐shell nanocrystals (NCs) showing excellent performance for both MOR and EOR. The structure, composition and size of the NCs can be controlled by adjusting the synthesis parameters in a one‐pot microwave‐assisted process. The electrocatalytic performance of the NCs shows lower potentials for both MOR and EOR compared to the OER. They consist of a copper‐rich metallic core initially encapsulated by a shell composed of cobalt oxide and cobalt carbide. This nanoheterostructure evolves to a copper oxide core surrounded by an oxide shell consisting of small cobalt‐ and copper‐oxide nanodomains upon chronopotentiometry experiments. The excellent performance in both MOR and EOR is attributed to the oxidation of the NCs and a concomitant diffusion process that forms small oxide clusters. The final structure provided NCs with high mass activities for both alcohol oxidation reactions, producing formic and acetic acid as products (for MOR and EOR, respectively). Finally, the NCs are tested for hybrid water electrolysis, demonstrating high hydrogen production along with high stability.

## Graphical abstract

Cu/Co‐based core‐shell nanocrystals (NCs) enable efficient hybrid water splitting via methanol and ethanol oxidation (MOR and EOR), outperforming traditional oxygen evolution reaction (OER). Synthesized via a one‐pot microwave process, the NCs evolve into oxide clusters that deliver high electrocatalytic activity and stability, enabling effective hydrogen production and yielding formic and acetic acids from MOR and EOR, respectively.

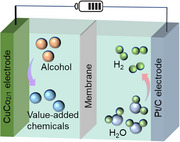

## 1 Introduction

Electrochemical water splitting is widely acknowledged as one of the most environmentally friendly processes to produce green hydrogen, especially when powered by renewable energy sources.^[^ ^1^, ^2^ ^]^ Nevertheless, the multiple electron transfer for the oxygen evolution reaction (OER) at the anode leads to a large energy barrier, limiting the kinetics of water's overall splitting.^[^ ^3^, ^4^ ^]^ To circumvent this issue, the sluggish OER can be replaced by a thermodynamically more favorable oxidation reaction, the so‐called hybrid water electrolysis.^[^ ^5^, ^6^ ^]^

Molecules such as alcohols, amines, urea, hydrazine, or carbohydrates have been studied intensively as excellent candidates for cost‐efficient and environmentally friendly production of hydrogen.^[^ ^7^, ^8^, ^9^ ^]^ Among them, methanol and ethanol stand out as excellent fuels for hybrid water electrolysis and direct alcohol fuel cells due to their high energy densities (17.3 and 22.7 MJ·L^−1^, respectively), ease of fuel handling, and low cost.^[^ ^10^, ^11^ ^]^ Both methanol and ethanol oxidation reactions (MOR and EOR, respectively) have been extensively studied to identify oxidation pathways that can lead to the total or partial oxidation of alcohol. While complete oxidation of both alcohols delivers the highest current densities (6 and 12 electrons for MOR and EOR, respectively), it also produces CO_2_, which increases the carbon footprint of the overall hydrogen production. In contrast, the partial oxidation of methanol or ethanol allows for the concomitant production of hydrogen and value‐added molecules under zero carbon footprints. In particular, formic acid follows the CO‐free oxidation pathway in the MOR, and acetic acid follows the C2 pathway in the EOR.^[^ ^12^ ^]^ In this context, ethanol emerges as a superior candidate for sustainable hydrogen production compared to methanol, owing to its higher energy density, lower toxicity, and renewable production from biomass.^[^ ^13^ ^]^

During hybrid water splitting, the applied voltages must remain below the required for the OER, thereby constraining the operating current densities and subsequently reducing the overall electrochemical efficiency.^[^ ^14^, ^15^ ^]^ This reduction in the current density is largely offset by the simultaneous production of hydrogen and value‐added molecules with zero CO_2_ emissions. For instance, by exploiting the electro‐oxidation of organic compounds, Yu et al. reduced the energy costs in ≈14.3%.^[^ ^16^ ^]^ Nevertheless, the electrocatalytic performance still requires improvements to achieve industrially viable Faradaic efficiencies and to attain current densities at a moderate applied potential. For the MOR, but particularly for the EOR, this is achieved by noble metal‐ (Pt‐ or Pd‐) and nickel‐based catalysts, which raises additional environmental and economic concerns.^[^ ^17^, ^18^, ^19^ ^]^ For instance, Qin et al. synthesized PtRuNiCoFeGaPbW high entropy alloy ultrathin nanowires to oxidate methanol to CO_2_.^[^ ^20^ ^]^ In these circumstances, transition metal‐based NCs (Cu, Co, and Ni) have been identified as potential electrocatalysts for alcohol oxidation reactions, offering an environmentally friendly and economically sustainable hydrogen production.^[^ ^21^ ^]^

For instance, Cu‐based materials show a high affinity to adsorb methanol and hence high activity for the MOR on Cu‐based NCs has been reported.^[^ ^22^ ^]^ Simultaneously, cobalt‐based catalysts have attracted much attention for their excellent performance in water splitting, in particular for the OER and MOR.^[^ ^19^, ^23^, ^24^ ^]^ Interestingly, cobalt doping in Ni‐, Pt‐ and Pd‐based catalysts results in an enhancement of the catalytic activity.^[^ ^25^, ^26^ ^]^

The combination of copper and cobalt in a NC has garnered significant attention due to the enhancement of the catalytic activity. Recently, various Cu‐Co nanomaterials, such as Cu@CoO_x_,^[^ ^27^ ^]^ CuCo_2_O_4_,^[^ ^28^ ^]^ copper cobalt phosphides,^[^ ^29^ ^]^ copper cobalt selenide,^[^ ^30^ ^]^ copper cobalt layered double hydroxide,^[^ ^31^ ^]^ boron‐copper cobalt oxides,^[^ ^32^ ^]^ copper cobalt hydroxycarbonates,^[^ ^33^ ^]^ or copper cobalt sulfide,^[^ ^34^ ^]^ have been reported.

The formation of Mott‐Schottky heterostructures has shown promising results in improving the intrinsic conductivity of oxide semiconductors.^[^ ^35^ ^]^ This approach induces interface effects in catalysts, such as electronic coupling, charge transfer and defects, and optimizing the heterostructure's intermediate adsorption/desorption behavior.^[^ ^36^ ^]^ The increased active site availability results in a superior electrocatalytic performance. Moreover, exploiting the Mott‐Schottky heterojunction interface between metal and semiconductor can significantly enhance semiconductor Fermi level management, utilizing Schottky barriers to achieve rectifying effects and confining semiconductor hole or electron states, thereby significantly improving electrocatalytic performance.^[^ ^37^ ^]^ Additionally, transition metal carbides are potential alternatives to platinum‐based catalysts, leveraging their platinum‐like electronic structures. Furthermore, synthesizing highly dispersed carbides as core nanoparticles supports synergistic enhancements.^[^ ^38^ ^]^ Recent literature underscores that synthesizing Mott‐Schottky heterostructures typically involves multistep syntheses, rendering them complex and time‐consuming.^[^ ^36^, ^39^, ^40^, ^41^ ^]^ Hence, there is an urgent need to explore a more rapid and streamlined synthesis route.

In this work, we report a one‐pot microwave (MW)‐assisted synthesis for producing Cu/Co‐based nanoheterostructures. These heterostructures, consisting of a metal core surrounded by a metal‐oxide/metal‐carbide shell, are embedded or self‐supported on a metal‐oxide sheet. Adjusting the feeding molar ratio between the copper and cobalt precursors provides control over the compositions of the core/shell NCs and the morphology of supporting metal‐oxide nanosheets. For instance, an excess of cobalt precursor leads to the formation of a CuCo alloy at the core and cobalt carbide and cobalt oxide domains at the shell. Under an applied potential, the structure evolves due to the diffusion and partial oxidation of atoms, resulting in an oxidized core surrounded by small Cu‐ and Co‐oxide domains. This process boosts the formation of Mott–Schottky heterojunctions along the structure, providing high electrocatalytic performance for both MOR and EOR. The nanoheterostructures were tested for hybrid water splitting, showing high stability under moderate current densities and the production of value‐added products such as formic or acetic acid.

## 2 Results and Discussion

### 2.1 Synthesis and Characterization of Cu/Co NCs

**Figure 1:**
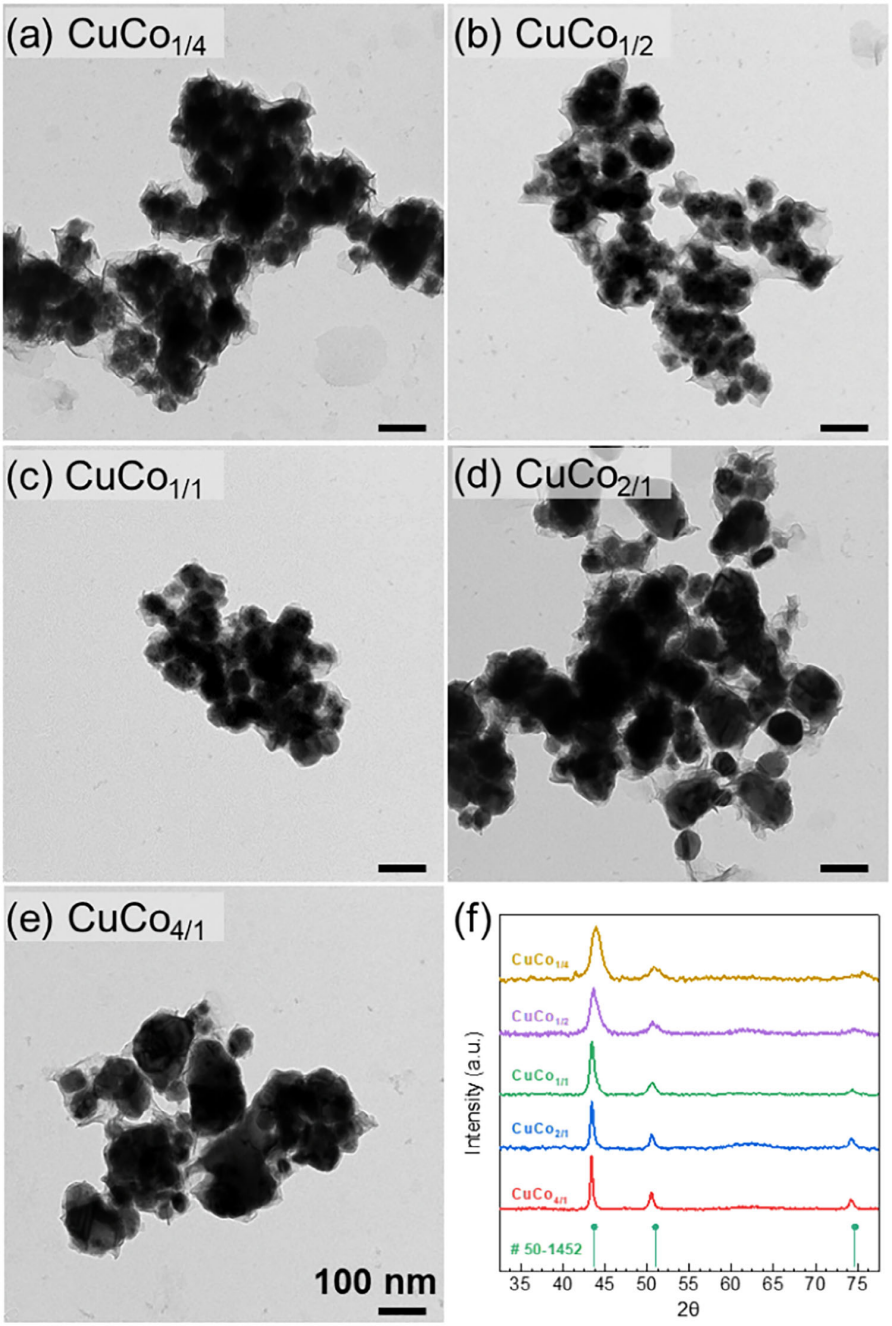
TEM images of Cu/Co NCs produced by a MW‐assisted route at 200 °C adding different Cu:Co feeding molar ratios: a) 1:4 – CuCo_1/4_, b) 1:2 – CuCo_1/2_, c) 1:1 – CuCo_1/1_, d) 2:1 – CuCo_2/1_, e) 4:1 – CuCo_4/1_, f) XRD patterns of Cu/Co NCs along with the reference for Cu_0.48_Co_0.52_ alloy (JCPDS: #50‐1452). All the scale bars correspond to 100 nm.

Self‐supported Cu/Co NCs were produced by a novel MW‐assisted synthesis route developed in this work. In particular, different molar ratios of copper‐ and cobalt‐acetylacetonates (i.e., Cu:Co ranging from 1:4 to 4:1) were mixed in a benzyl alcohol solution containing PVP_3.5k_. In all the experiments, the total metal concentration in the solution was kept constant at 17.5 mM. After MW radiation, nanoheterostructures consisting of NCs (regions with higher electron density) formed on top of thin substrates (areas of lower electron density) were observed (**Figure** **1**). Despite these nanoheterostructures varied in size according to the Cu:Co ratio, we could not identify a clear trend allowing us for a precise size control. Nevertheless, nanoheterostructures produced at 1:4 and 4:1 molar ratios show the largest cores (Figure 1a,e, respectively). According to the XRD results, all samples showed peaks consistent with the Fm‐3m(225) structure of Cu_0.48_Co_0.52_ (JCPDS #50‐1452). However, for the [111] and [200] peaks, we observed a deviation that was accentuated, in particular, for CuCo_1/4_ and CuCo_1/2_ samples, with peaks shifting toward higher angles as cobalt content increased (Figure S1, Supporting Information). This is consistent with the differences between the [111] peak for copper (43.297°, JCPDS #04‐0836) and cobalt (44.216°, JCPDS #15‐0806). Additionally, the presence of a broad peak at ≈61° and a small peak at 41.5° for the CuCo_1/4_ sample suggested the presence of copper or cobalt oxides. These results are compatible with the formation of a metallic Cu_x_Co_y_ core, self‐supported by a copper‐ and/or cobalt oxide shell.

According to the literature, the formation of a metallic core was unexpected, as similar conditions led to the formation of metal oxides.^[^ ^42^ ^]^ Control experiments were carried out by adding only one of the metal precursors (1:0 and 0:1), leading to the formation of Cu_2_O and CoO NCs (Figure S2, Supporting Information). This observation indicates that neither the PVP_3.5k_, benzyl alcohol, nor the purging of the tube with Ar, did provide enough reductive conditions to produce metallic NCs. Nonetheless, it is worth noticing that the synthesis conditions promote the reduction of copper precursor from Cu^2+^ to Cu^+^, resulting in the formation of a Cu_2_O phase. Thus, the formation of a metallic core would result from a redox synergy reaction between copper and cobalt complexes under these conditions as has been reported for instance for CuNi NCs.^[^ ^43^ ^]^ Further characterization confirmed that a Cu‐rich metallic core is formed in all the samples while cobalt is distributed between the core and the shell (see discussion below).

To further understand the formation of the Cu/Co NCs, the final stoichiometry of the samples was analyzed by ICP‐OES (Figure S3, Supporting Information). According to the results, we systematically observed a slightly higher copper content compared to the initial Cu:Co feeding ratios. For instance, the final copper contents for CuCo_1/4_ (20.0%), CuCo_1/2_ (33.3%), CuCo_1/1_ (50.0%), CuCo_2/1_ (66.6%) and CuCo_4/1_ (80.0%) were 23.1, 36.2, 55.8, 71.3 and 82.0%, respectively.

### 2.2 Electrocatalytic Characterization of Cu/Co NCs

**Figure 2:**
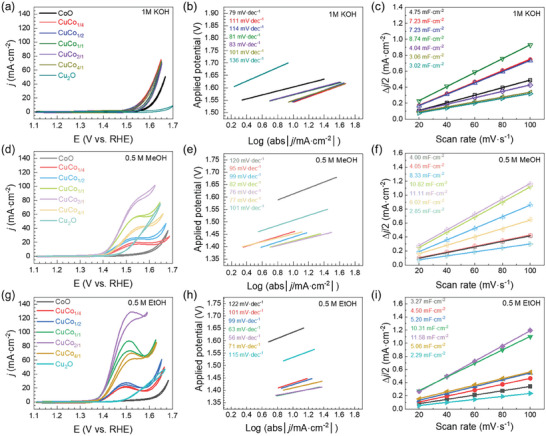
CV curves, Tafel and C_dl_ plots for all Cu/Co NCs samples measured in a‐c) 1 M KOH – OER; d‐f) in 1 M KOH + 0.5 M methanol – MOR and g‐i) in 1 M KOH + 0.5 M ethanol – EOR. Measurements were carried out by drop‐casting 5 µL of the catalyst ink in a glassy carbon electrode (WE). All potentials were corrected to the RHE and 90% of iR compensation. Note that Tafel plots (b, e and h) were plotted as a function of the applied potential.

The electrocatalytic performances of the Cu/Co NCs for the OER, MOR and EOR were evaluated at room temperature using a conventional three‐electrode system. Samples produced at 0:1 and 1:0 ratios (labeled as CoO and Cu_2_O, respectively) were also tested for a consistent analysis. All the measurements were carried out in a 1 M KOH electrolyte (OER), where methanol or ethanol (0.5 M) was added for the MOR and EOR (see Experimental Section for details). The summary of the CV polarization curves along with Tafel and C_dl_ plots are reported in **Figure** **2**.

#### 2.2.1 Cu/Co NCs Performance for the OER

Cu_2_O and CoO samples show the lowest OER activity among all samples (Figure 2a‐c). In particular, the Cu_2_O exhibited an onset potential above 1.60 V and overpotentials of 470 mV at 10 mA∙cm^−2^, the highest Tafel slope (136 mV∙dec^−1^) and the lowest C_dl_ value (3.02 mF∙cm^−2^). This performance obtained for the copper oxide NCs is consistent with previously published studies.^[^ ^44^ ^]^ CoO NCs' performance slightly improves with a lower overpotential (370 mV at 10 mA∙cm^−2^) and lower Tafel slope (79 mV∙dec^−1^). Yet, those values were still high compared to the substantial improvement in OER activity observed for Cu/Co NCs prepared with both copper and cobalt, where curves practically overlap for all samples. A closer look revealed that the Cu/Co nanoheterostructures produced at the highest cobalt contents showed the best performances (CuCo_1/4_ and CuCo_1/2_), with CuCo_1/2_ achieving the lowest overpotentials (310 and 390 mV at 10 and 50 mA∙cm^−2^, respectively). For comparison, the CuCo_2/1_ sample showed slightly higher overpotentials (340 and 410 mV at 10 and 50 mA∙cm^−2^, respectively). This suggests that increasing cobalt content enhances the OER efficiency, which is in good agreement with the superior OER performance of CoO relative to Cu_2_O alone. Despite the significant improvement of the Cu/Co NCs compared to the metal‐oxide NCs, these values remain comparable to those thus far reported in the literature for similar bimetallic nanoparticles.^[^ ^45^, ^46^ ^]^

#### 2.2.2 Cu/Co NCs Performance for the MOR

The addition of methanol to the electrolyte (0.5 M) resulted in a significant modification of the polarization curves (Figure 2d‐f). On one hand, the presence of methanol clearly improved the activity for the Cu_2_O NCs, displaying an onset potential at 1.46 V, an applied potential of 1.50 V at 10 mA∙cm^−2^, and a reduced Tafel slope (101 mV∙dec^−1^). Conversely, CoO NCs showed lower performance in the presence of methanol, as evidenced by higher overpotentials and steeper Tafel slopes than those measured for the OER. On the other hand, also in this case in all the Cu/Co NCs the performances improved with respect to the metal‐oxide NCs, exhibiting lower onset potentials (below 1.40 V vs RHE) and lower required potentials, along with a notable oxidation peak observed at ≈1.54 V versus RHE, characteristic of the occurrence of the MOR. According to the CV curves, activity increased with the increasing amount of copper in the NCs, reaching a peak performance for the CuCo_2/1_ sample. This sample showed the lowest applied potentials (1.42 and 1.48 V at 10 and 50 mA∙cm^−2^, respectively) and lower Tafel slope (76 mV∙dec^−1^), along with the highest oxidation peak current density (81.45 mA∙cm^−2^ at 1.55 V vs RHE) and the largest ECSA (277.75 cm^2^∙mg^−1^). This trend was opposite to the one observed for the OER where performances improved with increasing content of Co. Remarkably, the CuCo_1/1_ sample showed similar performance as the CuCo_2/1_ at lower potentials, though its performance decay as the potential increases, delivering lower current densities (1.42 and 1.51 V vs RHE at 10 and 50 mA∙cm^−2^, respectively, Figure 2d).

**Figure 3:**
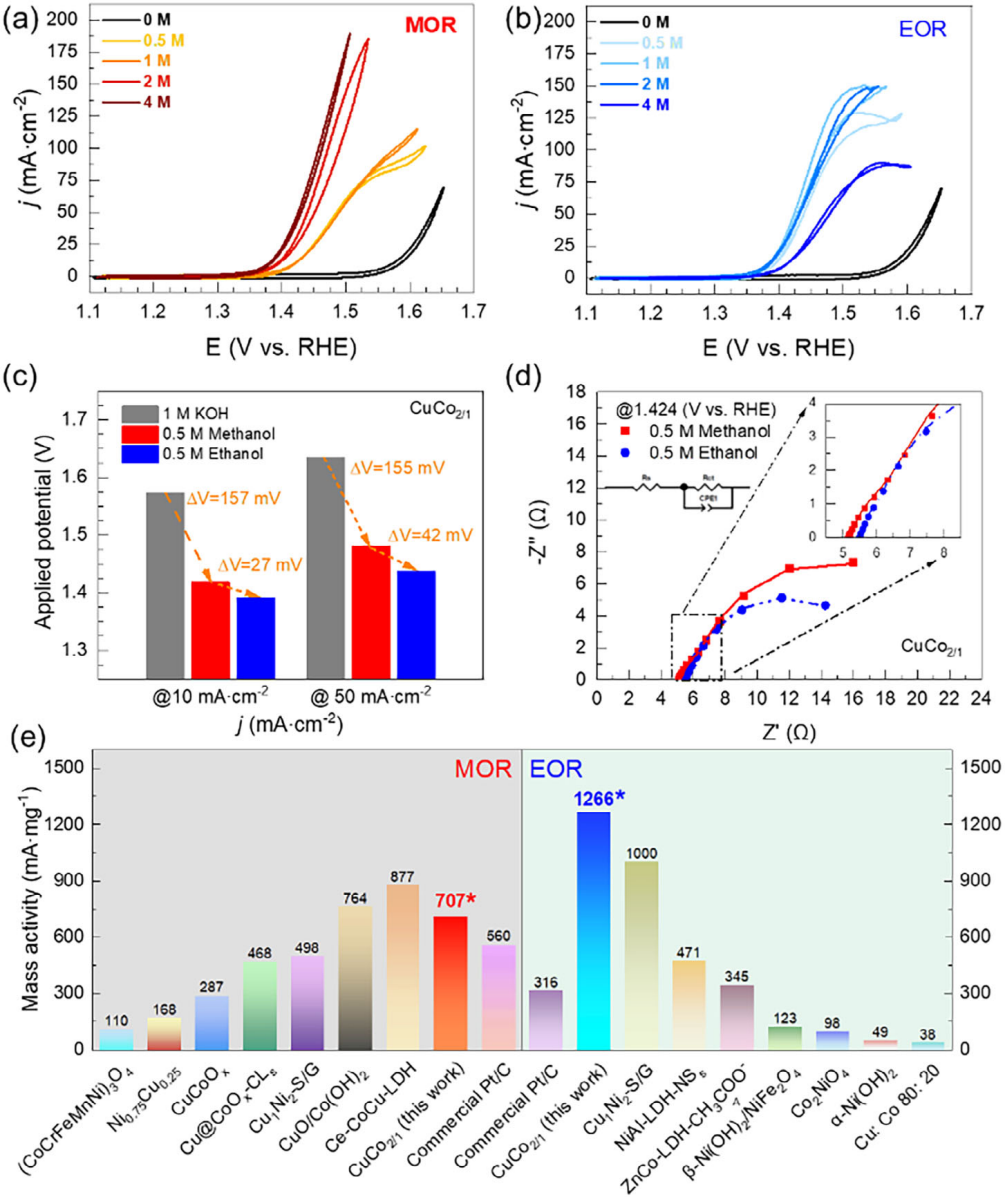
CV curves for the MOR a) and EOR b) at different methanol or ethanol concentrations (0, 0.5, 1, 2, 4 M). c) comparison of the potentials applied for CuCo_2/1_ NCs to reach 10 and 50 mA∙cm^−2^ in the OER, MOR, EOR. d) EIS for the CuCo_2/1_ in a KOH electrolyte containing 0.5 M methanol (red squares) or 0.5 M ethanol (blue circles). e) the comparison of mass activity for CuCo_2/1_ with the state of art for MOR and EOR based on transition metal‐based materials and commercial Pt/C (for references see Figure S6, Tables S1 and S2, Supporting Information).

Following the preliminary analysis, we studied the effect of the methanol concentration on the electrocatalytic performance of the CuCo_2/1_ NCs. CV curves were recorded at different methanol concentrations (0, 0.5, 1, 2, 4 M, **Figure** **3**a). Results showed a significant decrease in onset potential as the methanol concentration increased. At the same time, the oxidation peak at ≈1.54 V versus RHE gradually vanished, shifting from 1.55 to 1.40 V versus RHE. This observation clearly indicates an optimal methanol concentration for our experimental set‐up, where a trade‐off between the oxidation and diffusion rates is accomplished at the WE interface. At low concentrations, the reaction kinetics is likely limited by the diffusion of methanol molecules to the electrode surface. Instead, at higher concentrations, the methanol concentration and the oxidation products likely saturate the electrode's surface, thus decreasing the activity and, hence, the intensity of the oxidation peak.

#### 2.2.3 Cu/Co NCs Performance for the EOR

Finally, samples were tested for the EOR by adding 0.5 M ethanol into the electrolyte. Compared to methanol, ethanol is a non‐toxic compound that can be produced from biomass or agricultural feedstocks as well as waste. However, it has been well‐established that the presence of a higher carbon content results in lower oxidation efficiency.^[^ ^18^ ^]^ Indeed, we observed higher potentials at 10 mA∙cm^−2^ for both oxides during the EOR (1.52 and 1.63 V at 10 mA∙cm^−2^ for Cu_2_O and CoO, respectively) compared to the ones for MOR (1.46 and 1.61 V at 10 mA∙cm^−2^ for Cu_2_O and CoO, respectively). Certainly, this difference was particularly pronounced in the Cu_2_O sample, which exhibited higher values than the CoO. This indicates that cobalt demonstrates superior performance compared to copper, in contrast with the better performance of Cu_2_O for the MOR.

When analyzing the EOR activity of the Cu/Co NCs, all samples exhibited lower onset potentials compared to Cu_2_O and CoO, reflecting a similar trend to that observed for the MOR. The electrooxidation activity increased as the content of copper in the NCs increased, reaching its maximum for CuCo_2/1_ NCs. In particular, this sample showed an onset potential of 1.37 V along with required potentials as low as 1.39 and 1.44 V to reach current densities of 10 and 50 mA∙cm^−2^, respectively. Noteworthy, these values are even lower than those measured for the same sample but for the MOR (e.g., potentials of 1.42 and 1.48 V at 10 and 50 mA∙cm^−2^, respectively). Moreover, we observed that the ethanol oxidation peak was more pronounced, taking place at a lower voltage, and delivering higher current densities (i.e., 108.02 mA∙cm^−2^ at 1.50 V vs RHE for the EOR, compared to 81.45 mA∙cm^−2^ at 1.55 V vs RHE for MOR). These values clearly surpass those observed for the MOR and the OER, unequivocally identifying the CuCo_2/1_ NCs as a superior catalyst for the EOR. Indeed, we observed that at equal applied voltages, all NCs delivered higher current densities for the EOR compared with the MOR, underlining the effectiveness of these particular nanoheterostructures in catalyzing ethanol oxidation.

Similar to the MOR, we studied the effect of ethanol concentration by increasing the concentration from 0.5 to 4 M (Figure 3b). We observed a clear activity enhancement as the concentration increased to 1 M. However, further increasing the concentration of ethanol did not result in an increase in activity. Indeed, at a 4 M ethanol concentration, the current density decreased by almost a factor of 1.5 at high voltages (above ca. 1.45 V). This trend suggests the presence of intermediates that remain strongly absorbed by the active sites, reducing ECSA and thereby diminishing efficiency. For instance, an increasing production rate of acetic acid would eventually lead to a local increase of its concentration at the WE interface, displacing the equilibrium and hence decreasing the kinetic of the reaction.

#### 2.2.4 Comparison of the electrocatalytic performance for Cu/Co NCs

For a straightforward comparison of the OER, MOR, and EOR performance, we focused on the CuCo_2/1_ and compared the applied potentials at 10 and 50 mA∙cm^−2^. For instance, at 10 mA∙cm^−2^, the applied potentials for the EOR and the MOR were 157 and 184 mV lower compared to the OER. When the current density increased to 50 mA∙cm^−2^, the gap between the OER and MOR potentials was kept relatively constant (155 mV), whereas that for the EOR further increased to 197 mV. Likewise, the difference between MOR and EOR performances increased from 27 at 10 mA∙cm^−2^ to 42 mV at 50 mA∙cm^−2^. The EIS plots for MOR and EOR revealed that the solution resistance (R_s_) was comparable (Figure 3d; Table S3, Supporting Information) for both reactions, with MOR exhibiting similar R_s_ (5.14 Ω compared to 5.48 Ω for EOR). Additionally, the charge transfer resistance (R_ct_) for ethanol oxidation (11.73 Ω) was lower than that for methanol oxidation (16.78 Ω), indicating that CuCo_2/1_ has a lower overall resistance, providing a faster kinetics for ethanol oxidation, which is in agreement with the observed higher activity for the EOR.

Finally, the mass activities of the CuCo_2/1_ NCs and commercial Pt/C for the MOR and EOR at 50 mA∙cm^−2^ were directly compared using the same experimental set‐up along with the data reported in the literature for transition metal‐based catalysts (Figure 3e). For the MOR, CuCo_2/1_ NCs exhibited slightly higher mass activities compared to commercial Pt/C (707 against 560 mA·mg^−1^) and comparable to recently reported values on cobalt−copper layer double hydroxide and CuO/Co(OH)_2_ nanosheets (877 mA∙mg^−1^ and 764 mA∙mg^−1^, respectively).^[^ ^31^, ^47^ ^]^ Notably, commercial Pt/C exhibits a peak at low applied voltages (0.78 V vs RHE, Figure S6, Supporting Information). However, the relatively low magnitude of this peak significantly limits its overall catalytic activity, as increasing the voltage beyond this point leads to a decline in current density. On the other hand, CuCo_2/1_ NCs exhibited a rather high mass activity for EOR (1266 mA∙mg^−1^). which is significantly higher compared to similar reported works for Cu_1_Ni_2_‐S/G (1000 mA∙mg^−1^)^[^ ^48^ ^]^ or commercial Pt/C catalyst (316 mA∙mg^−1^). Hence, CuCo_2/1_ NCs showed remarkable mass activities for both alcohol oxidation reactions, similar to or outperforming state‐of‐the‐art Cu‐Co‐based catalysts. This observation is of particular interest as best performing catalysts for MOR typically do not show high activities for EOR, and vice versa. Overall, CuCo_2/1_ NCs exhibited dual catalytic activity for both ethanol and methanol oxidation, demonstrating exceptional high performance in the more challenging EOR. These findings prompted a more in‐depth characterization of CuCo_2/1_ NCs to investigate the origin of this enhanced performance.

### 2.3 Characterizations of CuCo_2/1_ NCs

**Figure 4:**
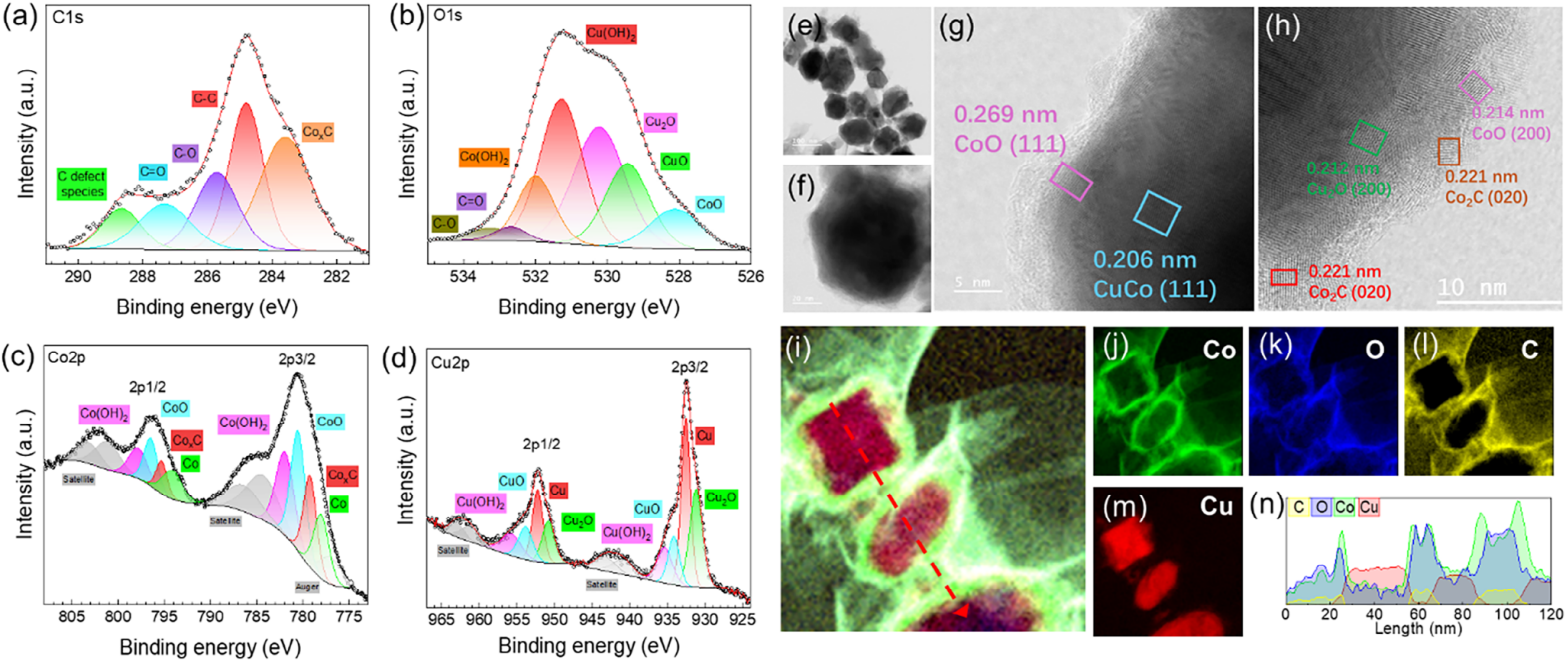
a) C1s, b) O1s, c) Co2p, and d) Cu2p XPS high‐resolution spectra of CuCo_2/1_. e,f) Low magnification and g,h) HR‐TEM images of CuCo_2/1_ NCs. i‐m) EELS spectrum image of CuCo_2/1_, and Co *L*‐, O *K*‐, C *K*‐, and Cu *L*‐edge elemental maps, respectively. n) elemental profile along different NCs as shown by the arrow in cobalt carbide.

The structure of the CuCo_2/1_ NCs was further elucidated through XPS analysis (**Figure** **4**a–d; Figure S7, Supporting Information). Deconvolution of the C1s high‐resolution spectrum revealed five components with binding energies at 288.7, 287.3, 285.7, 284.8, and 283.6 eV, corresponding to O─C═O, C═O, C─OH, C─C, and metal carbide, respectively (Figure 4a). The carbide signal was later correlated to cobalt carbide by analyzing the cobalt 2p high‐resolution energy spectrum. The cobalt spectrum was deconvoluted in up to twelve peaks (excluding an LMM Auger peak at 777.0 eV, Figure 4c), with the peaks at 779.2 and 795.3 eV corresponding to the 2p_3/2_ and 2p_1/2_ orbitals of cobalt carbide, respectively. The presence of four satellite peaks at 784.3, 788.4, 801.1, and 803.2 eV associated with the cobalt carbide and Co^2+^ peaks, confirmed the presence of the carbide phase.^[^ ^49^ ^]^ Additional peaks were fitted to metallic cobalt (778.0, 794.1 eV), CoO (780.5, 796.5 eV), and Co(OH)_2_ (781.9, 797.8 eV). Similarly, the Cu2p XPS spectrum (Figure 4d) revealed twelve peaks, including those for Cu_2_O (931.2 and 950.8 eV), metallic copper (932.6 and 952.3 eV), CuO (934.2 and 953.9 eV), and Cu(OH)_2_ (935.4 and 955.8 eV), along with satellite peaks associated with CuO (945.0, 943.3, 961.2 and 963.0 eV). The deconvolution of the O 1s spectra (Figure 4b) corroborated the presence of copper and cobalt oxides and hydroxides (CuO – 528.1 eV; CoO – 529.4 eV; Cu_2_O – 530.2 eV; Cu(OH)_2_ – 531.3 eV; Co(OH)_2_ – 532.0 eV), along with C═O and C─O peaks (532.7 and 533.3 eV, respectively). The confirmation by XPS of a carbide phase was unexpected due to the preliminary XRD data and the synthesis conditions. Nonetheless, it has been reported the direct synthesis of Co_2_C and Co_3_C nanoparticles by the polyol processes as well as the presence of cobalt carbide in the synthesis of metallic cobalt nanoparticles.^[^ ^50^ ^]^

In order to study the morphology and distribution of the different phases observed by XPS, HR‐TEM and EELS analyses were conducted (Figure 4e‐n). TEM images clearly reveal a core‐shell structure, with a core ≈50 to 100 nm in diameter surrounded by a 5 to 10 nm thick shell. The measured d‐spacing at the core (0.206 nm) matched with the [111] direction of a metallic CuCo structure, while the d‐spacing at the shell (0.269 nm) agreed with the [111] direction of a CoO structure (Figure 4g). This confirmed the presence of a metallic core surrounded by a metal oxide shell. As previously mentioned, the core‐shell domains are supported on a thin and heterogeneous nanosheet. The analysis of the area corresponding to the nanosheet revealed the presence of crystallographic planes compatible with Cu_2_O (0.212 nm, [200]), CoO (0.214 nm, [200]), and Co_2_C (0.221 nm, [020]) structures. This confirms the coexistence of multiple crystallographic phases within the NCs, suggesting the presence of different heterojunctions (likely a Mott‐Schottky junction) that may serve as catalytic active sites, thereby enhancing the catalytic activity.^[^ ^51^ ^]^

The distribution of copper and cobalt was further studied by acquiring EELS and EDX spectrum images of the NCs (Figure 4i‐n; Figure S8, Supporting Information). The EELS elemental maps shown in Figure 4i‐n reveal that Cu‐rich areas are surrounded by Co, C, and O regions. EELS analyses performed in different regions of the self‐supported NCs corroborated the concentration of copper at the cores along with traces of cobalt and oxygen in the shell (see Figure S9, Supporting Information). Noteworthy, the blunt Cu *L*‐edge peak indicates the presence of metallic copper at the core of the NCs,^[^ ^52^ ^]^ whereas the shell (blue square in Figure S9, Supporting Information) shows a decrease in the copper signal and a significant increase of the Co, C, and O signals. In particular, the presence the O signal and the sharpness of the Co *L*‐edge indicate the presence of cobalt oxide spices.

In summary, CuCo_2/1_ NCs exhibit a core‐shell structure with a Cu‐rich metallic core coated by a shell composed of a mixture of copper and cobalt oxides, along with a Co_2_C phase. It is important to note that the presence of small amounts of cobalt within the core cannot be entirely ruled out. As for the support, CoO and Co₂C phases extend outward to form the core‐shell structure, forming a nanosheet that serves as support. Although CoO and Co₂C were the predominant phases identified through various characterization techniques, a minor presence of copper was detected by EELS mapping. This unique structure comprises the presence of metal‐semiconductor and semiconductor‐semiconductor heterojunctions, which eventually may lead to an enhancement of the catalytic activity of the core due to the accumulation of charge at the interface.^[^ ^35^ ^]^ Indeed, catalysts comprising a heterojunction have been successfully employed for hydrogen production.^[^ ^53^, ^54^ ^]^ In our particular case, the additional synergy between the core‐shell domain and the nanosheet could ultimately boost the efficiency of the self‐supported NCs.

### 2.4 Alcohol‐Assisted Water Electrolysis

**Figure 5:**
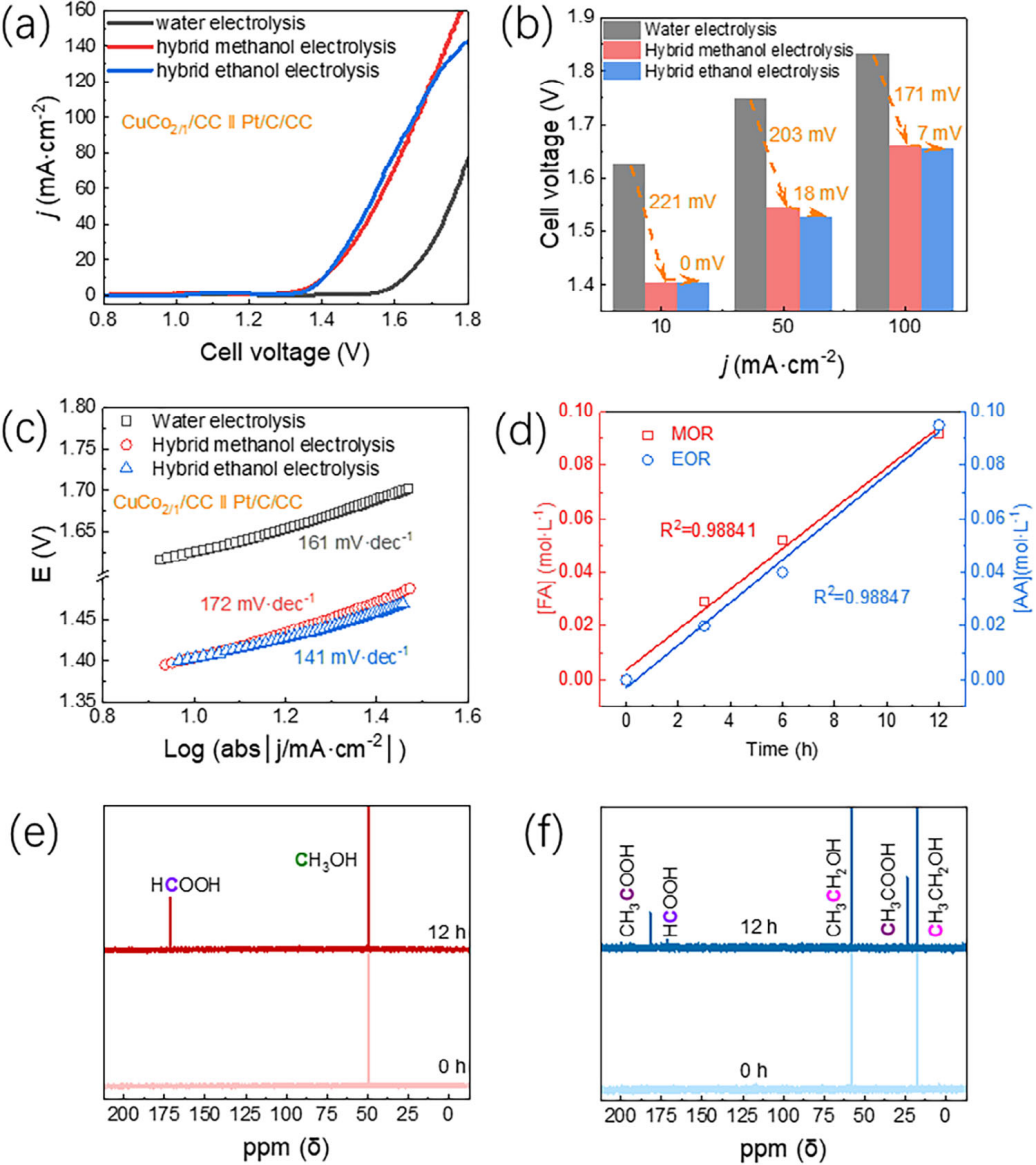
a) LSV polarization curves of conventional water electrolysis (1 M KOH), alcohol‐assisted water splitting (1 M KOH + 1 M and 1 M KOH + 1 M ethanol) for CuCo_2/1_/CC║Pt/C/CC system. b) required cell voltage comparison between conventional and alcohol‐assisted water electrolysis at 10, 50, and 100 mA∙cm^−2^, respectively. c) Tafel slopes, d) quantification of fitting curves for formate (FA) / acetate (AA) products during the methanol or ethanol assisted water electrolysis. e) ^13^C NMR analysis of products for methanol assisted water electrolysis for 12 h under 40 mA∙cm^−2^. f) ^13^C NMR analysis of products for ethanol assisted water electrolysis for 12 h under 40 mA∙cm^−2^.

To demonstrate the applicability of the CuCo_2/1_ catalyst for hybrid water electrolysis, a two‐electrode system was set up where the anode and the cathode were functionalized with CuCo_2/1_ NCs and commercial Pt/C, respectively. Both electrodes were prepared by drop‐casting the catalyst inks onto carbon cloth (CuCo_2/1_/CC and Pt/C/CC electrodes). For a comparative evaluation, the CuCo_2/1_/CC║Pt/C/CC system was tested in three different electrolytes (KOH, KOH + Methanol and KOH + Ethanol). First, in 1 M KOH solution (conventional water splitting, **Figure** **5**a), the onset potential was ≈1.58 V. Current densities of 10, 50, and 100 mA∙cm^−2^ were achieved under an applied potential of 1.63, 1.75, and 1.83 V, respectively, with a Tafel slope of 161 mV∙dec^−1^. Adding methanol to the electrolyte (1 M KOH and 1 M methanol, hybrid methanol‐assisted water splitting) reduced significantly the applied potentials at 10, 50, and 100 mA∙cm^−2^ (ΔV = 221, 203 and 171 mV, respectively, Figure 5b). Finally, a similar behavior was observed at lower voltages by adding ethanol (1 M KOH and 1 M ethanol, ethanol assisted water electrolysis). In particular, the onset and applied cell potential at low current density (10 mA∙cm^−2^) are comparable to methanol‐assisted water electrolysis (1.38 and 1.40 mV, respectively, Figure 5a,b). However, the applied potentials decreased at higher current density compared to methanol (ΔV = 18 and 7 mV for 50 and 100 mA∙cm^−2^, respectively). So did the Tafel slope, showing the lowest value for ethanol among all three electrolytes (141 mV∙dec^−1^, Figure 5c). This observation aligns with the better EOR performance observed for the CuCo_2/1_ NCs (Figure 3c), highlighting it as the most promising candidate for hybrid water splitting, simultaneously producing hydrogen along with value‐added molecules.

To investigate the reaction products of the aforementioned process, an H‐type electrolytic cell was used to separate the alcohol oxidation reaction from the hydrogen evolution reaction while preventing the reduction of products formed from the MOR and the EOR. The products were analyzed via proton and carbon NMR spectroscopy (Figure 5e,f; Figure S10, Supporting Information). In the ^13^C NMR spectrum for the methanol‐assisted water electrolysis, a peak at 171.59 ppm appeared after 12 h, indicating the presence of formate in coexistence with methanol (49.50 ppm).^[^ ^55^ ^]^ To evaluate the product concentration over time, ^1^H NMR spectra were conducted at different time intervals (0, 3, 6, and 12 h), using maleic acid as the internal standard (6.00 ppm, shifting to 6.31 ppm Figure S10b, Supporting Information). The chemical shift observed in Figure S10 (Supporting Information) is in line with the conversion of maleic acid to fumaric acid via cis–trans isomerization.^[^ ^56^ ^]^ Quantitative analysis was performed by integrating the peak areas, resulting in a constant formate production over reaction time (Figure 5d). From these observations, we calculated the Faradaic efficiency for methanol‐to‐formate conversion, which was found to be 82.2%. This high efficiency underscores the catalyst's effectiveness in selectively producing formate as the primary reaction product.

In the context of ethanol‐assisted water electrolysis, after 12 h, we observed two peaks corresponding to ethanol at 17.49 and 58.07 ppm, along with two additional peaks at 23.92 and 182.03 ppm, which can be attributed to the two different carbon environments of acetate (Figure 5f).^[^ ^57^ ^]^ Additionally, we observed a small peak at 171.61 ppm, indicating the presence of formate, suggesting that acetate is the primary product of ethanol oxidation, accompanied by a minor amount of formate as a byproduct. This observation is further supported by ^1^H NMR results where peaks corresponding to acetate and formate are observed at 1.89 ppm and 8.63 ppm, respectively (Figure S10c,d, Supporting Information). The concentration of acetate was determined following the same procedure as in the methanol‐assisted water electrolysis (blue line in Figure 5d). From this data, the selectivity for formate and acetate was 6.1% and 66.7%, respectively, resulting in a total Faradaic efficiency of 91.0% (83.3% for acetate and 7.7% for formate, respectively). Overall, the Faradaic efficiencies for MOR and EOR are comparable to the representative nanocatalysts reported in the literature, as listed in Table S4 (Supporting Information).

Based on the above discussion, we propose a rational reaction pathway for the EOR under our experimental conditions. In this mechanism, ethanol is initially oxidized to generate the key adsorbed intermediate, *CH_3_CHO (Figure S11, Supporting Information). This intermediate predominantly follows a pathway leading to the formation of acetic acid as the main product. Eventually, a fraction of this acetic acid may follow further oxidation delivering formic acid.^[^ ^58^, ^59^ ^]^ This is in well, agreement with the traces of the later found in the NMR spectra. However, a small fraction of *CH₃CHO may follow alternative routes, resulting in the formation of acetaldehyde or volatile gaseous products such as CO or CO_2_. The generation of these minor byproducts may partially account for the observed decrease in FE. Further investigation, including more sensitive detection techniques such as gas chromatography or in situ spectroscopic analysis combined with DFT simulations is required to fully elucidate the reaction pathways and quantify the complete product distribution. However, this is beyond the scope of the present publication.

**Figure 6:**
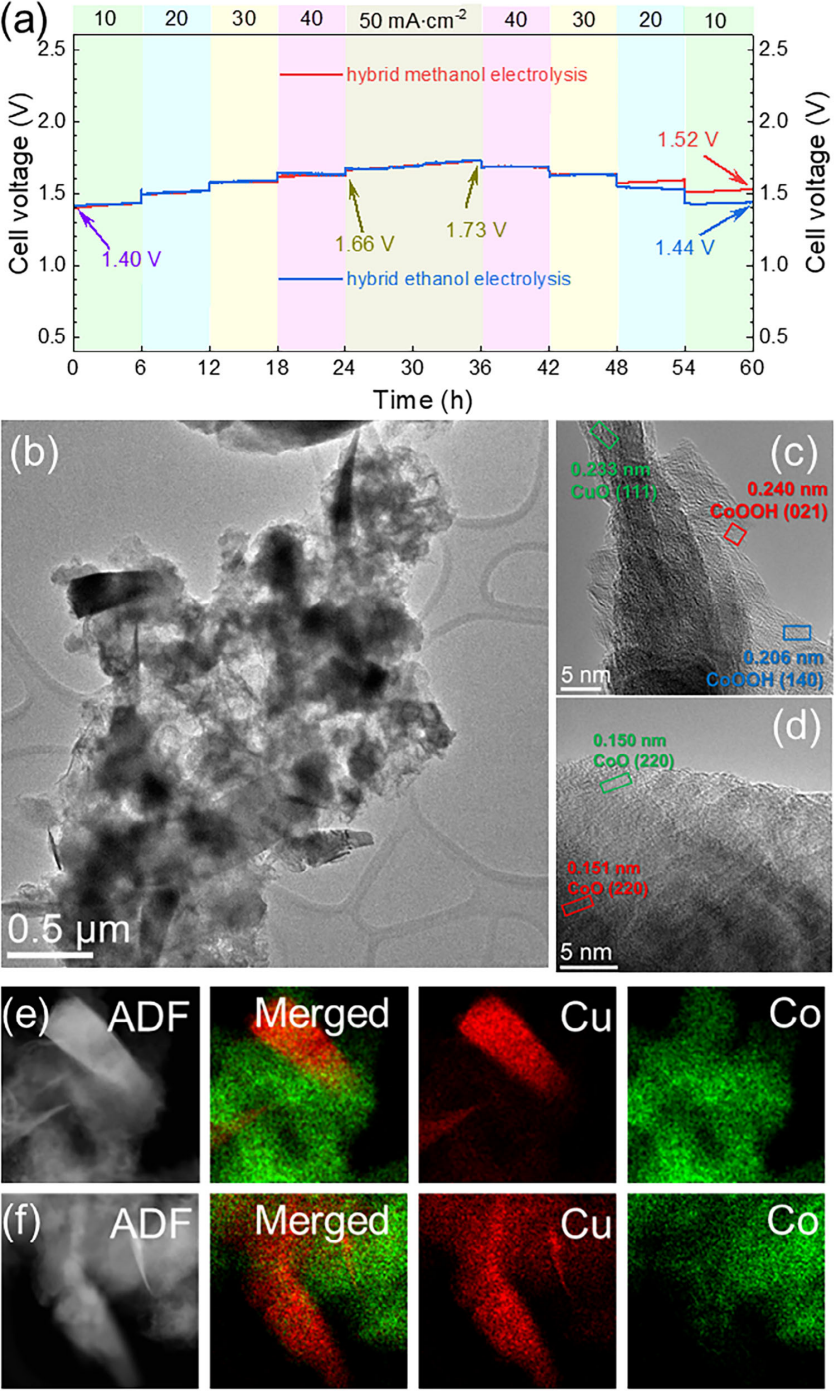
a) Chronopotentiometry of CuCo_2/1_/CC║Pt/C/CC system in 1 M KOH + 1 M methanol (red line) and 1 M KOH + 1 M ethanol (blue line). b) TEM image of the CuCo_2/1_ samples after 60 h chronopotentiometry for ethanol‐assisted water ethanol electrolysis. c,d) HR‐TEM images focusing on NCs and NPs in Figure 6b, respectively. e,f) EDX mapping of the CuCo_2/1_ sample after chronopotentiometry test.

Finally, we studied the stability of the CuCo_2/1_ catalyst by performing multistep chronopotentiometry experiments at different current densities (10, 20, 30, 40, and 50 mA∙cm^−2^). The setup was immersed in a 1 L electrolyte solution containing 1 M KOH and 1 M methanol or ethanol to ensure an adequate alcohol supply. The current was incrementally raised every 6 h up to 50 mA·cm^−2^, where it was kept for 12 h before decreasing it at the same rate, leading to an overall measurement of 60 h (**Figure** **6**a). For the methanol‐assisted water electrolysis, the cell voltage increased gradually at each constant current density, with a minimal increase at low current densities (e.g., from 1.40 to 1.43 V at 10 mA·cm^−2^ after 6 h), and a more pronounced increase at higher current densities, reaching 1.73 V at 50 mA∙cm^−2^ after 12 h.

This difference is clearly observed when considering the slope of the V versus t curve in Figure 6a for each current density (Table S5, Supporting Information). When decreasing the current back to 10 mA·cm^−2^, the voltage remained almost constant at high current densities (40 and 30 mA·cm^−2^) but slightly increased at low current densities. For instance, from 1.57 to 1.59 V (48 and 53 h; 30 mA·cm^−2^) or from 1.50 to 1.52 V (54 to 60 h; 10 mA·cm^−2^). Indeed, after 60 h, the final voltage did experience an increase of 0.13 V with respect to the voltage at the same current density but at time zero. The same study was conducted for ethanol‐assisted water electrolysis providing a similar trend. The initial potential of 1.40 V at 10 mA∙cm^−2^ gradually increased over time, with the applied current density reaching 1.73 V at 50 mA∙cm^−2^ after 60 h (see Table S5, Supporting Information). Nevertheless, when decreasing the applied current density, the cell voltage remained almost constant at any step, delivering a final cell voltage of 1.44 V at 10 mA∙cm^−2^. This represents an increase with respect to the cell voltage at time zero of 0.04 V (ca. 2.8%). This underlines the better stability of the NCs for the ethanol‐assisted water electrolysis.

The voltage increase over time could be attributed to different factors, such as a rapid consumption of alcohol molecules in solution (methanol or ethanol) during the reaction or the transformation of the NCs. By comparing the LSV curves of the NCs before and after the multistep chronopotentiometry in methanol and ethanol, we observed a significant shift of the curve at higher voltages for the MOR, while for EOR, the curves were maintained (Figure S12a,d, Supporting Information). In order to elucidate the influence of alcohol consumption against catalyst transformation, after the chronopotentiometry the electrolyte was replaced by a fresh solution and additional 6 h at 10 mA·cm^−2^ was carried out (Figure S12b,e, Supporting Information). Interestingly, the initial potential for methanol was higher than the initial potential at the start of the multistep chronopotentiometry (1.53 V against 1.40 V) after electrolyte replacement. Conversely, for ethanol, the potential remained consistent (1.41 V). After 6 h at constant current densities, the cell potential increases (1.56 and 1.44 V for methanol and ethanol, respectively). From this observation, we conclude that methanol‐assisted water electrolysis did have a strong inference in the catalyst structure, while for ethanol, the addition of fresh electrolyte seems to restore the performance completely. This trend was supported by EIS analysis, where the R_ct_ for methanol increased from 0.75 Ω up to 3.85 Ω after the chronopotentiometry. Remarkably, after replacing the electrolyte, the R_ct_ further increased to 8.49 Ω. The same trend was observed for the mass transfer resistance (R_mt_) of MOR. As for the EOR, the increases in both resistances were moderate but clearly far below those increases observed for MOR. In particular, the R_mt_ values increased within the same order of magnitude (104 to 351 Ω). Noteworthy, by replacing the old electrolyte with a fresh one, the resistances for both alcohol oxidation reactions increased. This clearly indicates that the increases measured for the cell voltages during the chronopotentiometry tests are rather related to the evolution of the catalyst's structure.

A deeper understanding of the structural evolution of the core‐shell self‐supported nanostructures was obtained by analyzing CuCo_2/1_ NCs after the chronopotentiometry. As shown in Figure 6, NCs underwent a significant morphological transformation after the long‐term stability test in ethanol, resulting in the formation of self‐supported large and elongated nanostructures. These elongated domains show d‐lattice spacings corresponding to the [111] plane of CuO, which confirmed the oxidation of the pristine copper core. The shell of the NCs also evolved, resulting in the formation of CoOOH ([021] and [140] planes) and CoO ([220] plane) phases. EDX and EELS elemental mapping of the CuCo_2/1_ NCs confirmed the redistribution of elements along the NCs (Figure 6e,f; Figure S12, Supporting Information). Noteworthy, we observed that areas containing both cobalt (778 and 792 eV) and copper (929 and 950 eV) oxides were surrounded by areas in which only one of the oxides was present. The energies and shape of the spectra were in agreement with the oxidation state of cobalt and copper, confirming that the core underwent an oxidation process. This particular configuration would corroborate the outstanding performance of the NCs as CuO domains are surrounded by CoO and CoOOH ones. In particular, the latter have been identified as a true active species for OER, likely acting as active sites boosting the electrocatalytic activity of CuO.^[^ ^60^ ^]^ XPS analysis of the NCs after the EOR stability test further supports our findings (Figure S13, Supporting Information). A detailed deconvolution of the Co 2p energy region was performed to resolve overlapping components assigned to CoO (780.5 eV, 795.5 eV) and CoOOH (782.3 eV, 797.1 eV). Compared to the spectra of the as‐synthesized NCs, the metallic cobalt signals are no longer observed, indicating their transformation. Furthermore, part of the Co^2+^ species is oxidized to CoOOH. Similarly, deconvolution of the Cu 2p region reveals peaks corresponding to metallic Cu (932.7 eV, 952.1 eV), CuO (934.8 eV, 954.3 eV), and CuOOH (936.3 eV, 956.4 eV). While the metallic Cu signal remains detectable, it is significantly diminished, and the emergence of CuOOH indicates partial oxidation of Cu^2+^ to higher‐valence states. These findings are consistent with the EELS characterization discussed above, confirming a structural evolution in which Co and Cu atoms diffuse from the metallic core toward the formation of heterojunctions. A similar structure evolution was observed for NCs after chronopotentiometry in the presence of methanol (Figures S14 and S15, Supporting Information).

## 3 Conclusion

We successfully developed a complex yet ordered bimetallic Cu/Co Mott‐Schottky nanohybrid structure using a simple and rapid one‐pot microwave‐assisted method. This structure features a metallic Cu/Co core, surrounded by a mixture of copper oxide, cobalt oxide, and cobalt carbide phases extending into nanosheets providing support for the Cu/Co core. The NCs evolve from the original Mott‐Schottky heterostructure into a CuO‐CoOOH‐CoO composite during electrolysis.

The resulting architecture features a rich array of heterogeneous interfaces, significantly enhancing the oxidation activity toward methanol and ethanol. The observed increase in activity can be attributed to the induced and enhanced self‐driven charge transfer within the heterogeneous structure, which balances the adsorption of reactants and the desorption of products. When employing these NCs as anode for alcohol‐assisted water electrolysis, potentials are clearly reduced compared to conventional water electrolysis while maintaining stability for over 60 h. Moreover, alongside the hydrogen evolution reaction, formic and acetic acids are produced at the anode, achieving high selectivity and Faradaic efficiency (91.0% for ethanol oxidation). These findings provide a novel experimental approach for synthesizing multi‐interface, high‐activity heterogeneous structures, offering additional strategies to address the high cell potential required for conventional water electrolysis and enhancing the overall value of the reaction process.

## 4 Experimental Section

### Materials

Cobalt(II) acetylacetonate (Co(acac)_2_, ≥ 99.9%), copper(II) acetylacetonate (Cu(acac)_2_, ≥ 99.9%), benzyl alcohol (for synthesis), potassium hydroxide (KOH, 85% ACS reagent), nafion 117 containing solution (≈5% in a mixture of low aliphatic) and 2‐propanol (≥ 99.5%) were purchased from Sigma–Aldrich. Polyvinylpyrrolidone (PVP_3.5k_, Mw = 3′500) was purchased from Thermo Fisher Scientific. Carbon black (Vulcan CX‐72) was purchased from FuelCellStore. All materials were used as received without further purification. Milli‐Q water (18.2 MΩ·cm, Milli‐Q IQ, Merck Millipore) was used for all the experiments and solutions. Commercial Pt/C catalyst (20wt% of Pt loading) was purchased from Quintech. Carbon cloth (W0S1101) was purchased from CeTech Co., Ltd.

### MW‐Assisted Synthesis of Cu/Co NCs

For the MW‐assisted synthesis of Cu/Co NCs, a MW reactor (CEM Discover SP) operating at a frequency of 2.45 GHz and a power of 300 W was used. Samples were produced by modifying a procedure for the synthesis of Cu_2_O NCs previously reported by the group.^[^ ^42^ ^]^ The amount of benzyl alcohol and PVP_3.5k_ were kept constant to 4 mL and 136 mg (0.038 mmol), respectively. The copper to cobalt molar feeding ratio was modified keeping a final metal concentration of 17.5 mM (Co + Cu). For instance, for a 2:1 copper to cobalt ratio, 136 mg of PVP_3.5k_ (0.038 mmol) were dissolved in 4 mL of benzyl alcohol through continuous sonication (20 min) in a 10 mL reaction tube. Next, 12.30 mg of Cu(acac)_2_ (0.047 mmol) and 5.90 mg of Co(acac)_2_ (0.023 mmol) were added into the reaction tube, and the solution was sonicated further until a homogeneous solution was formed (ca. 30 min). The final solution was then purged by bubbling with an Ar flow for at least 20 min and the tube was sealed with a rubber. Finally, the tube was placed inside the MW reactor, heated to 60 °C, and kept at that temperature for 5 min. The temperature was then raised up to 200 °C (ca. 180 seconds) and kept at 200 °C for 10 min before cooling down to room temperature with compressed air. The NCs were collected by adding 30 mL of acetone followed by centrifugation at 6000 rpm for 10 min. The supernatant was discarded and the procedure repeated at least 3 times before drying the precipitate under vacuum at room temperature. Based on the copper‐to‐cobalt molar ratio, the sample was labelled as CuCo_2/1_.

Samples with different copper to cobalt molar ratios were synthesized following the same procedure but adjusting the feeding Cu(acac)_2_ and Co(acac)_2_ ratios. In detail, the copper: cobalt feeding molar ratio was adjusted to 1:4, 1:2, 1:1 and 4:1 leading to the synthesis of the CuCo_1/4_, CuCo_1/2_, CuCo_1/1_, CuCo_2/1_, CuCo_4/1_ samples, respectively. Additionally, control samples using only Cu(acac)_2_ or Co(acac)_2_ (1:0 or 0:1 molar ratio) were synthesized. These samples were labeled as Cu_2_O (CuCo_1/0_) and CoO (CuCo_0/1_), respectively.

### NCs Characterization—NCs Characterization—X‐Ray Diffraction (XRD)

Patterns were collected using a conventional Cu‐Kα radiation source (λ = 1.5406 Å) in a Bruker ADVANCE D8 employing a Bragg–Brentano geometry. Intensity was recorded within the 2θ range from 20° to 80° at a scanning step of 0.02°·s^−1^. Samples were prepared by drop‐casting and further evaporating a concentrated solution of NCs onto an amorphous silicon wafer.

### NCs Characterization—Electron Transmission Microscopy (TEM) and High‐Resolution TEM (HR‐TEM) Analysis

Low‐resolution electron micrographs were obtained using a JEOL 1210 TEM operating at 120 kV, equipped with a side‐entry 60°/30° double tilt GATAN 646 specimen holder. High‐resolution TEM (HR‐TEM) images, high‐angle annular dark‐field scanning TEM (HAADF‐STEM) images, and electron energy loss spectroscopy (EELS) mapping were conducted by a Spectra 300 (S)TEM operated at 300 kV (Thermo Fisher). Samples were prepared by drop‐casting a diluted solution of NCs onto carbon‐coated Au‐grids or Cu‐grids (400 mesh).

### NCs Characterization—X‐Ray Photoelectron Spectroscopy (XPS)

XPS measurements were done in a SPECS PHOIBOS 150 hemispherical energy analyzer using Al Kα X‐ray radiation (1486.6 eV) for excitation.

### NCs Characterization—Inductively Coupled Plasma Atomic Emission Spectroscopy (ICP‐OES)

To determine the final copper to cobalt ratio in the NCs, as well as the yield of the reaction, ICP‐OES was carried out using an Agilent model 5900. Samples were prepared by digesting ≈1 mg of NCs in 1 mL of aqua regia for 30 min. The solution was diluted with Milli‐Q water reaching a final volume of 10 mL.

### Electrochemical Characterizations

The electrochemical performance of each sample was analyzed utilizing a Bio‐logic VSP multichannel potentiostat. A conventional three‐electrode cell configuration was employed where a 5 mm diameter glassy carbon disk was used as the working electrode (WE), a platinum mesh as the counter electrode (CE) and a Hg/HgO electrode as the reference electrode (RE). Cyclic voltammetry (CV) in 1 M KOH at a sweeping rate of 50 mV·s^−1^ was performed for each catalyst.

### Electrochemical Characterizations—Preparation of the catalyst

The NCs produced were supported onto carbon black to prepare the catalyst and evaluate their electrocatalytic activity. In detail, 4 mg of Cu/Co NCs and 2 mg of carbon black (2:1, mass ratio) were dispersed in 5 mL of ethanol through continuous sonication for 3 h to ensure a homogenous mixing. The suspension was then dried under vacuum at 60 °C for 12 h. Finally, 5 mg of the dried catalyst was dispersed in a 1 mL of solution (200 µL of Milli‐Q water, 700 µL of 2‐propanol and 100 µL of Nafion) and sonicated for 30 min until a homogenous ink was formed.

### Electrochemical Characterizations—Preparation of the WE

First, the surface of the electrode was polished with alumina powder until achieving a mirror‐like surface. Afterwards, 5 µL of the catalyst ink containing the Cu/Co NCs was drop‐casted onto the WE surface. All potentials were corrected to the reversible hydrogen electrode (RHE) according to the following equation: E_RHE_ = E_Hg/HgO_ + 0.059·pH + 0.098 V, where the pH was determined by a pH meter.

### Electrochemical Characterizations—OER, MOR and EOR Characterization

For evaluating the performance of the samples for OER, MOR and EOR, CV curves were acquired in a KOH solution containing different concentrations of methanol or ethanol (0, 0.5, 1, 2, and 4 M). Tafel slopes were determined from Tafel plots following the equation: η = b · log*j* + c, where b is the Tafel slope, η is the overpotential and *j* is the current density. Electrochemical double‐layer capacitance (C_dl_) curves were measured by running CV curves in the non‐Faradaic region (1.174–1.274 V vs RHE) at different scan rates (from 20 to 100 mV·s^−1^). The C_dl_ was calculated by following the equation: C_dl_ = Δ*j*/v, where Δ*j* = (*j*_a_‐*j*_c_)/2) is the current density (at 1.224 V vs RHE, mA·cm^−2^) and v is the scan rate (mV·s^−1^). The electrochemical active surface area (ECSA) was calculated as: ECSA = C_dl_/C_s_, where a general specific capacitance (C_s_) of 0.04 mF·cm^−2^ in 1 M KOH was used.^[^ ^61^ ^]^ Electrochemical impedance spectroscopy (EIS) was acquired with an amplitude of 5 mV at frequencies ranging from 10^6^ to 10^−2 ^Hz. Finally, the mass activity (MA) of the catalyst was calculated according to the following equations: MA = *j*/m, where *j* is the measured current density (mA·cm^−2^) and m is the mass of NCs. Partial iR compensation (90%) was applied to all data manually.

### Electrochemical Characterizations—Alcohol‐Assisted Water Electrolysis

Conventional and hybrid water electrolysis were evaluated using a two‐electrode configuration employing a HCl‐activated carbon cloth (CC) (1 × 1.5 cm^2^) as both anode and cathode. Electrodes were prepared by drop‐casting a solution containing as‐prepared CuCo_2/1_ NCs (anode) or commercial Pt/C (cathode). For instance, 5 mg of CuCo_2/1_ NCs were dispersed in a solution containing 200 µL of MQ‐H_2_O, 700 µL of 2‐propanol, and 100 µL of Nafion under 3 h of sonication. Next, 200 µL of this solution was drop casted onto the CC to achieve a loading of 1 mg∙cm^−2^. The anode was labeled as CuCo_2/1_/CC. The same procedure was used to prepare the cathode, but the CuCo_2/1_ NCs were replaced by commercial Pt/C. In this case, 400 µL were drop cast onto the CC to achieve 2 mg·cm^−2^ (anode labeled as Pt/C/CC).

Linear sweep voltammetry (LSV) was applied to both anodes in a 1 M KOH electrolyte solution (conventional water splitting) and in a 1 M KOH electrolyte solution containing 1 M methanol and ethanol concentrations (hybrid alcohol electrolysis). Data were analyzed using the same equations described previously. Stability tests were carried out by running chronopotentiometry measurements at 10, 20, 30, 40, and 50 mA·cm^−2^ for 60 h for hybrid methanol‐ and ethanol‐water splitting.

### Electrochemical Characterizations—Characterizations of the Products

To characterize the byproducts of the alcohol‐assisted water electrolysis, the electrolyte was analyzed after chronopotentiometry measurements at 40 mA·cm^−2^ over time (0, 3, 6, 12 h) in an H‐type cell employing a Nafion 117 membrane. In detail, 0.3 mL of the electrolyte was mixed with 0.3 mL of D_2_O and ^13^C and ^1^H nuclear magnetic resonance (NMR) spectroscopies were carried out in a Bruker Avance III HD 400 MHz. For a quantitative analysis, maleic acid (0.1 mL) was used as an internal standard.^[^ ^55^ ^]^ The corresponding selectivity (S) and faradaic efficiencies (FE) for each reaction were calculated at a static current at different times using the following equations:

(1) $$S\left(\%\right)=\frac{N}{N_{OH}}\cdot 100;FE\left(\%\right)=\frac{\sum N_{i}\cdot 4F}{Q}\cdot 100$$

Where *N* is the moles of the as‐produced molecule, *N_OH_* is the moles of consumed alcohol (methanol or ethanol), *Q* is the total charge passed through the system recorded during electrolysis, and *F* is the Faraday constant (96485 C∙mol^−1^).

## Conflict of Interest

The authors declare no conflict of interest.

## Author Contributions

X.Z. performed conceptualization, investigation, data acquisition, formal analysis, wrote the original draft. J.G. performed investigation, formal analysis. A.P. performed formal analysis. D.T. performed Formal analysis, funding acquisition, writing‐review & editing. P.G. performed conceptualization, formal analysis, investigation, methodology, funding acquisition, project administration, supervision, wrote the original draft, writing‐review & editing.

## Supporting information



Supporting Information

## Data Availability

The data that support the findings of this study are available in the supplementary material of this article.
