# Supplementary material for: Microwave‐Assisted Synthesis of Cu/Co‐Based Nanoheterostructures for High‐Efficiency Alcohol Oxidation
Source: Adv Sci (Weinh). 2025 Jul 25;12(38):e05581. doi: 10.1002/advs.202505581 (PMC12520565; doi:10.1002/advs.202505581)
Supplement: Supplementary file 1 — Supporting Information [file ADVS-12-e05581-s001.docx]

**Microwave-Assisted Synthesis of Cu/Co-Based Nanoheterostructures for High-Efficiency Alcohol Oxidation**

Xuesong Zhang,^1^ Jaume Gázquez,^1^ Arturo Pajares,^2^ Dino Tonti,^1^ Pablo Guardia^1,*^

*^1)^ Institut de Ciència de Materials de Barcelona (ICMAB-CSIC), Campus UAB, Bellaterra 08193, Spain*

*^2)^ Materials & Chemistry, Flemish Institute for Technological Research (VITO NV), Boeretang 200, Mol 2400, Belgium*

**Table of contents:**

**Figure S1** (a) XRD pattern for CuCo_x/y_ NCs. (b) and (c) magnification of particular regions of the spectra where a peak shift is observed for samples CuCo_1/4_ and CuCo_1/2_.

**Figure S2** TEM images of (a) 0:1 – CoO (CuCo_0/1_), (b) 1:0 – Cu_2_O (CuCo_1/0_) produced by a microwave-assisted route at 200 °C. All the scale bars are corresponding to 100 nm. (c) XRD spectra for CuCo_1/0_ and CuCo_0/1_.

**Figure S3** Comparison between the feeding and real Cu content determined by ICP-OES.

**Figure S4** CVs measured at different scan rates (20 to 100 mV∙s^-1^) and different electrolytes (1 M KOH, 1M KOH +0.5 M methanol, and 1 M KOH + 0.5 M ethanol) for all the samples.

**Figure S5** CVs measured at different scan rates for CoO and Cu_2_O under 1 M KOH, 1M KOH +0.5 M methanol, and 1 M KOH + 0.5 M ethanol.

**Figure S6** The CV measurements for commercial Pt/C and comparison with as-synthesized CuCo_2/1_ for MOR (1 M KOH + 0.5 M MeOH) and EOR (1 M KOH + 0.5 M EtOH).

**Figure S7** The overview XPS spectra of CuCo_2/1_.

**Figure S8** EDX mapping of CuCo_2/1_ NCs (a: ADF, b: merged, c: carbon, d: oxygen, e: cobalt, f: copper).

**Figure S9** EELS C k, O k, Co L, Cu L profiles based on different positions. 1) green square: Domain on nanoparticle; 2 and 3) blue and red squares: Nanosheet areas; and 4) gray square: Carbon support.

**Figure S10** (a) ^1^H NMR analysis of products for hybrid methanol electrolysis for 12 h under 40 mA∙cm^-2^. (b) ^1^H NMR analysis of products for hybrid methanol electrolysis for 0, 3, 6, 12 h under 40 mA∙cm^-2^ with internal reference. (c) ^1^H NMR analysis of products for hybrid ethanol electrolysis for 12 h under 40 mA∙cm^-2^. (d) ^1^H NMR analysis of products for hybrid ethanol electrolysis for 0, 3, 6, 12 h under 40 mA∙cm^-2^ with internal reference. Insets in panel c and d reveal the presence of a small peak corresponding to formic acid.

**Figure S11** Schematic diagram of the possible ethanol oxidation pathway and the further oxidation of acetic acid to formic acid.

**Figure S12** (a) Comparison of CuCo_2/1_/CC║Pt/C/CC for hybrid methanol electrolysis before, after 60 h chronopotentiometry (Figure 6a), and after refreshing electrolyte. (b) Chronopotentiometry test at 10 mA∙cm^-2^ for 6 h after refreshing the electrolyte for hybrid methanol electrolysis. (c) the corresponding EIS plots of the time frame in Figure S12a. (d) the comparison of CuCo_2/1_/CC║Pt/C/CC for hybrid ethanol electrolysis before, after 60 h chronopotentiometry (Figure 6a), and after refreshing electrolyte. (e) chronopotentiometry test at 10 mA∙cm^-2^ for 6 h after refreshing the electrolyte for hybrid ethanol electrolysis. (f) the corresponding EIS plots of the time frame in Figure S12d.

**Figure S13** (a) EELS mapping, (b) profiles of Figure S13a at different positions.

**Figure S14** High-resolution XPS analysis of the (a) C1s, (b) O1s, (c) Co2p and (d) Cu2p energy regions for CuCo_2/1_ NCs after carrying a stability measurement for EOR (Figure 6).

**Figure S15** (a) TEM image of the CuCo_2/1_ samples after 60 h chronopotentiometry for hybrid methanol electrolysis measurement. (b), (c) HR-TEM images focusing on NCs and NPs in Figure S15a, respectively. (d), (e) EDX mapping of CuCo_2/1_ sample after chronopotentiometry test.

**Figure S16** (a) EELS mapping, (b) profiles of Figure S16a at different positions of sample CuCo_2/1_ after hybrid methanol electrolysis.

**Table S1** Mass activities of CuCo_2/1_ and the state of the art for MOR.

**Table S2** Mass activities of CuCo_2/1_ and the state of the art for EOR.

**Table S3** Values for the equivalent circuits of Figure 3d.

**Table S4** Comparison of Faradaic Efficiencies for MOR and EOR between CuCo_2/1_ NCs and representative nanocatalysts reported in the literature at different voltages.

**Table S5** Summary of potentials at different stages of the multistep chronopotentiometry in Figure 6a.

**Table S6** Values for the equivalent circuits of Figure S12c, f.

**References**

**Figure S1** (a) XRD pattern for CuCo_x/y_ NCs. (b) and (c) magnification of particular regions of the spectra where a peak shift is observed for samples CuCo_1/4_ and CuCo_1/2_.

**Figure S2** TEM images of (a) 0:1 – CoO (CuCo_0/1_), (b) 1:0 – Cu_2_O (CuCo_1/0_) produced by a microwave-assisted route at 200 °C. All the scale bars are corresponding to 100 nm. (c) XRD spectra for CuCo_1/0_ and CuCo_0/1_.

**
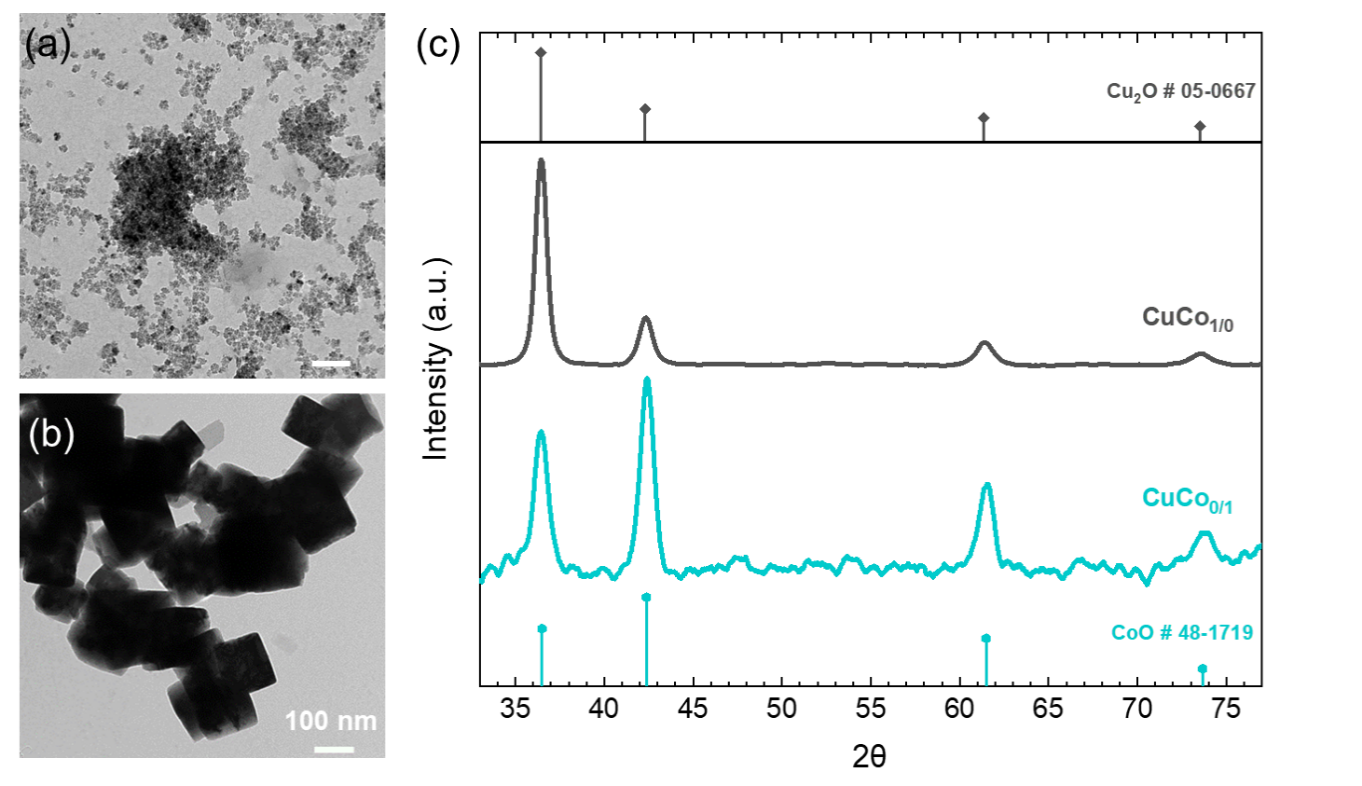
**

**Figure S3** Comparison between the feeding and real Cu content determined by ICP-OES.

**Figure S4** CVs measured at different scan rates (20 to 100 mV∙s^-1^) and different electrolytes (1 M KOH, 1M KOH +0.5 M methanol, and 1 M KOH + 0.5 M ethanol) for all the samples.


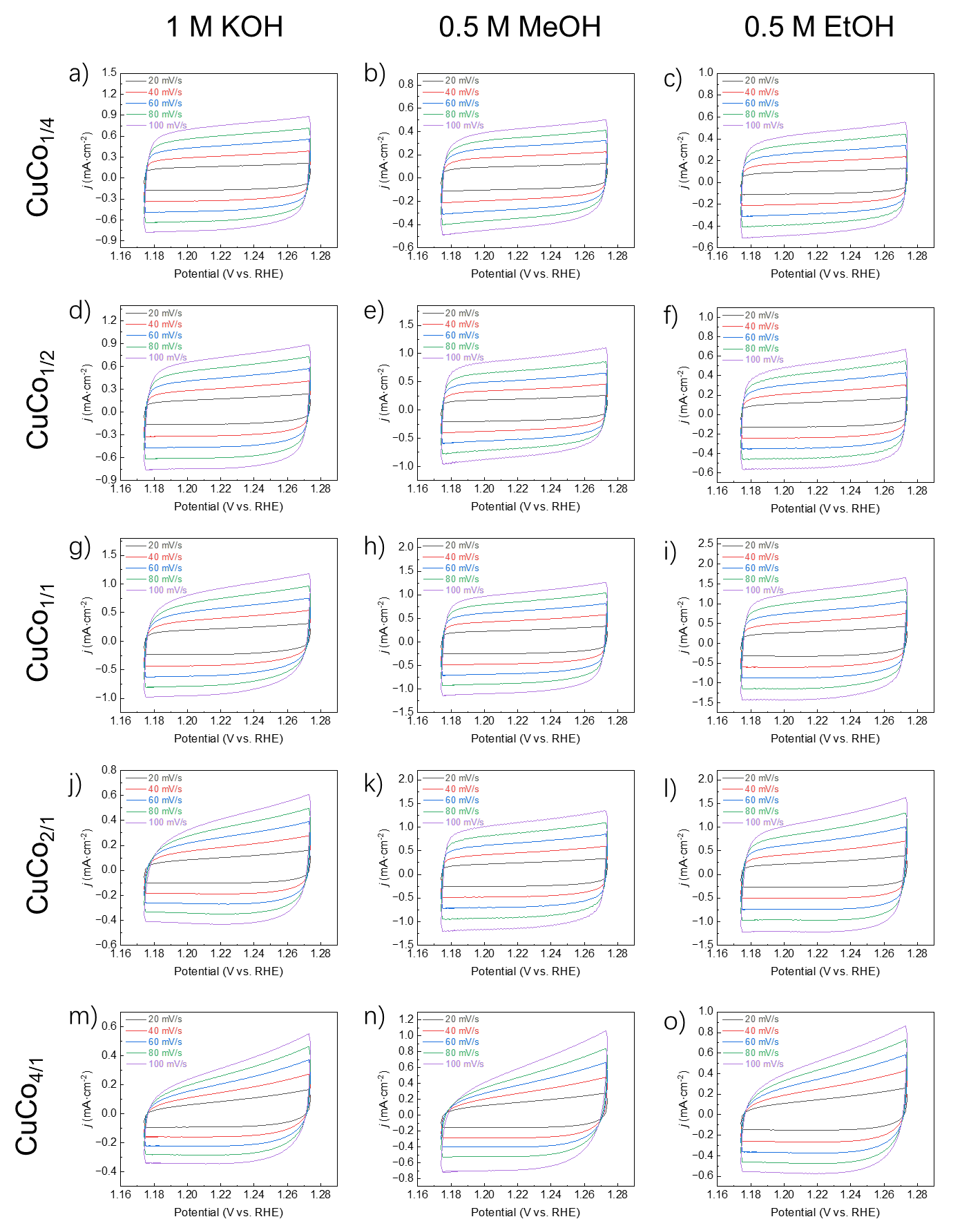


**Figure S5** CVs measured at different scan rates for CoO and Cu_2_O under 1 M KOH, 1M KOH +0.5 M methanol, and 1 M KOH + 0.5 M ethanol.


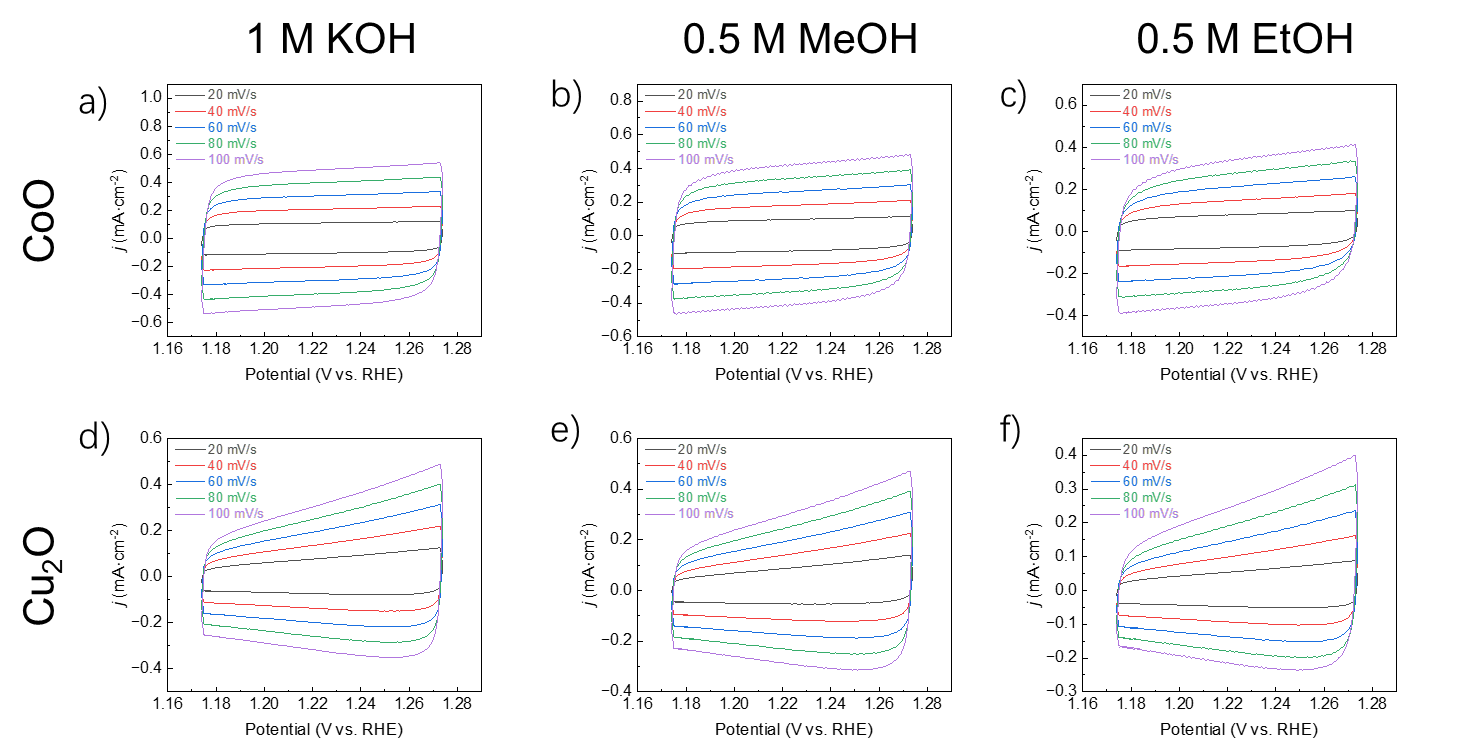


**Figure S6** The CV measurements for commercial Pt/C and comparison with as-synthesized CuCo_2/1_ for MOR (1 M KOH + 0.5 M MeOH) and EOR (1 M KOH + 0.5 M EtOH).

**Figure S7** The overview XPS spectra of CuCo_2/1_.

**Figure S8** EDX mapping of CuCo_2/1_ NCs (a: ADF, b: merged, c: carbon, d: oxygen, e: cobalt, f: copper).


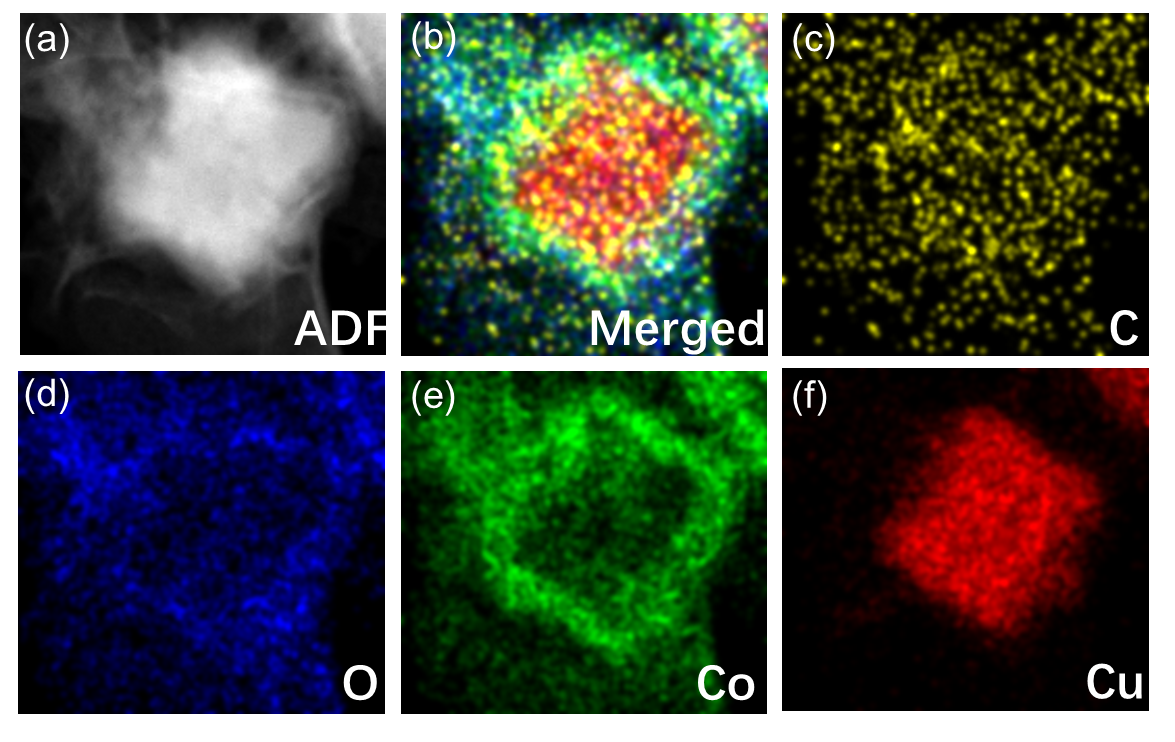


**Figure S9** EELS C k, O k, Co L, Cu L profiles based on different positions. 1) green square: Domain on nanoparticle; 2 and 3) blue and red squares: Nanosheet areas; and 4) gray square: Carbon support.


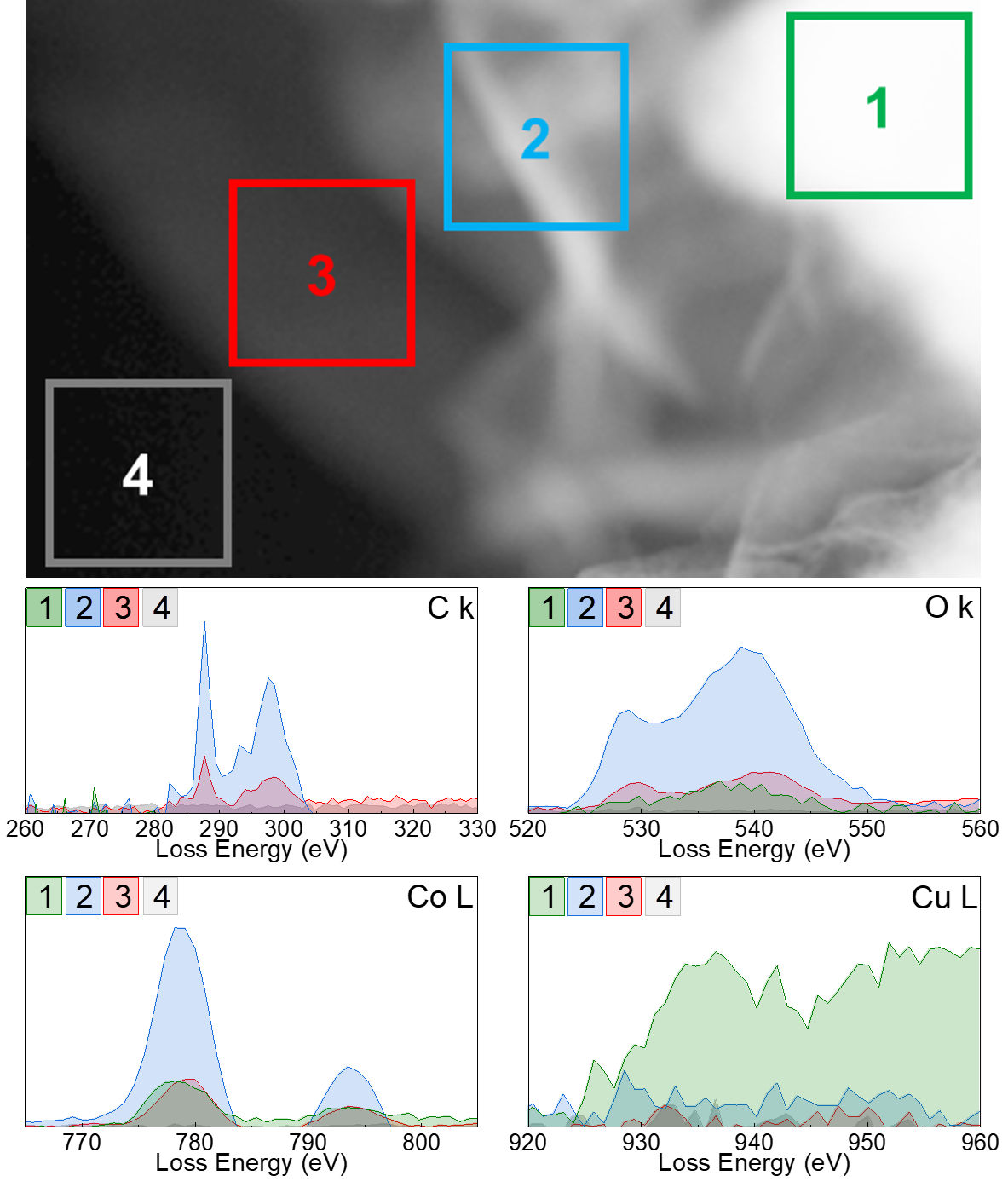


**Figure S10** (a) ^1^H NMR analysis of products for hybrid methanol electrolysis for 12 h under 40 mA∙cm^-2^. (b) ^1^H NMR analysis of products for hybrid methanol electrolysis for 0, 3, 6, 12 h under 40 mA∙cm^-2^ with internal reference. (c) ^1^H NMR analysis of products for hybrid ethanol electrolysis for 12 h under 40 mA∙cm^-2^. (d) ^1^H NMR analysis of products for hybrid ethanol electrolysis for 0, 3, 6, 12 h under 40 mA∙cm^-2^ with internal reference. Insets in panel c and d reveal the presence of a small peak corresponding to formic acid.

**Figure S11** Schematic diagram of the possible ethanol oxidation pathway and the further oxidation of acetic acid to formic acid.


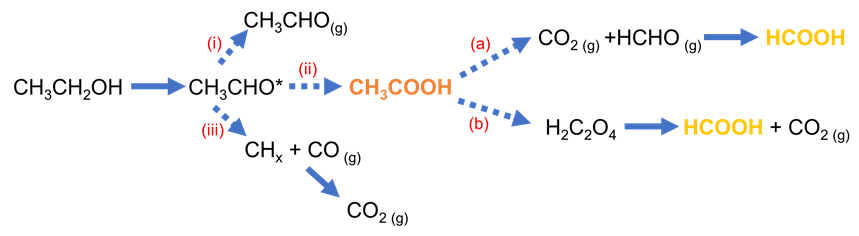


**Figure S12** (a) Comparison of CuCo_2/1_/CC║Pt/C/CC for hybrid methanol electrolysis before, after 60 h chronopotentiometry (Figure 6a), and after refreshing electrolyte. (b) Chronopotentiometry test at 10 mA∙cm^-2^ for 6 h after refreshing the electrolyte for hybrid methanol electrolysis. (c) the corresponding EIS plots of the time frame in Figure S12a. (d) the comparison of CuCo_2/1_/CC║Pt/C/CC for hybrid ethanol electrolysis before, after 60 h chronopotentiometry (Figure 6a), and after refreshing electrolyte. (e) chronopotentiometry test at 10 mA∙cm^-2^ for 6 h after refreshing the electrolyte for hybrid ethanol electrolysis. (f) the corresponding EIS plots of the time frame in Figure S12d.

**Figure S13** (a) EELS mapping, (b) profiles of Figure S13a at different positions.

**
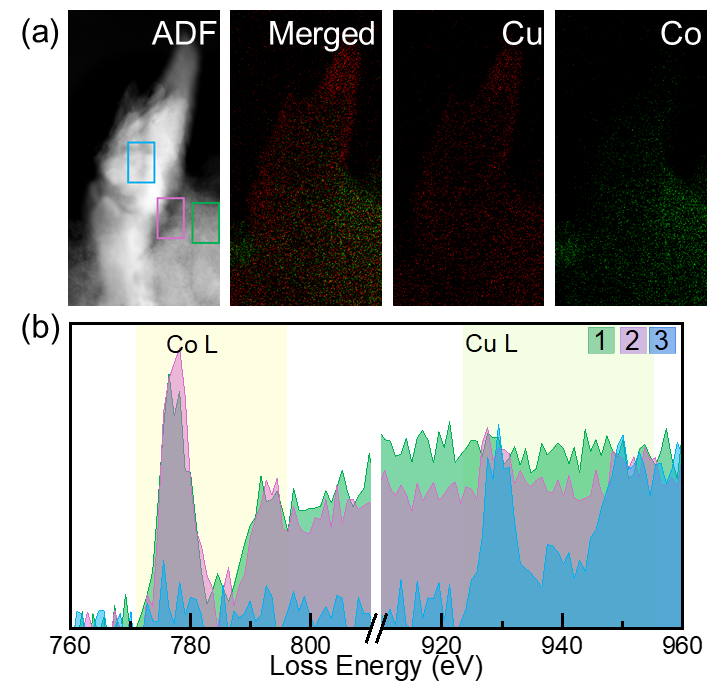
**

**Figure S14** High-resolution XPS analysis of the (a) C1s, (b) O1s, (c) Co2p and (d) Cu2p energy regions for CuCo_2/1_ NCs after carrying a stability measurement for EOR (Figure 6).

**
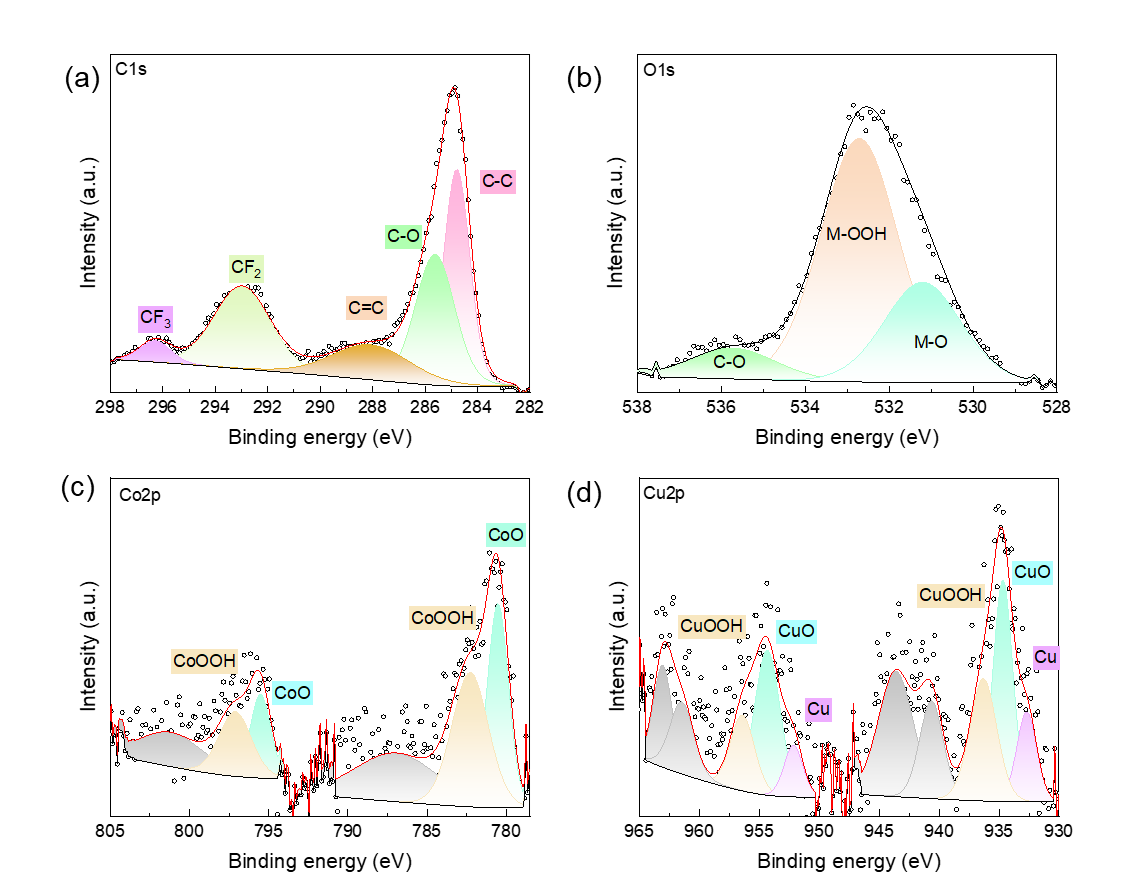
**

**Figure S15** (a) TEM image of the CuCo_2/1_ samples after 60 h chronopotentiometry for hybrid methanol electrolysis measurement. (b), (c) HR-TEM images focusing on NCs and NPs in Figure S15a, respectively. (d), (e) EDX mapping of CuCo_2/1_ sample after chronopotentiometry test.

**
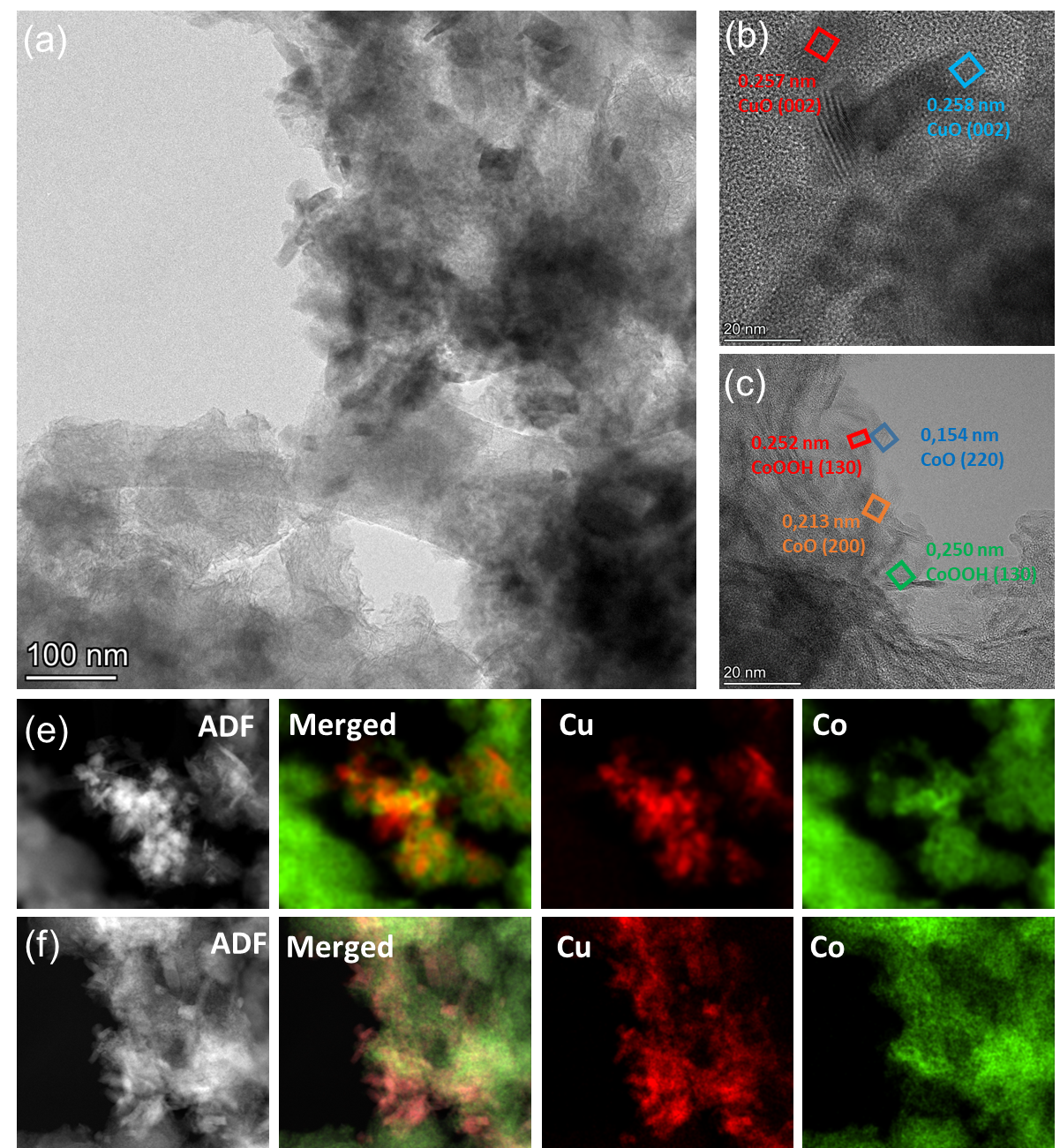
**

**Figure S16** (a) EELS mapping, (b) profiles of Figure S16a at different positions of sample CuCo_2/1_ after hybrid methanol electrolysis.

**
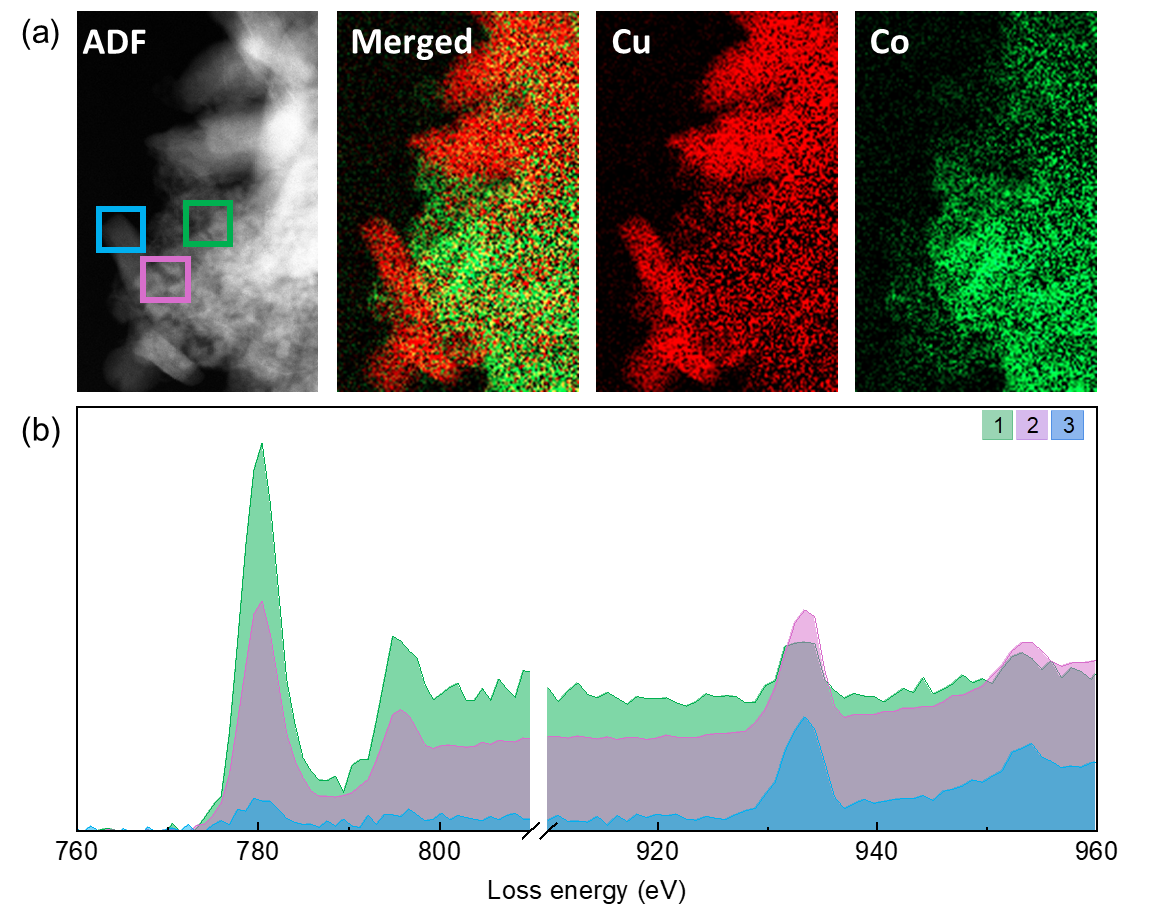
**

**Table S1** Mass activities of CuCo_2/1_ and the state of the art for MOR.

|  | **Sample** | **Mass activity (mA∙mg^-1^)** | **Potential (V vs. RHE)** | **Electrolyte** | | **Supporter** | **Reference** |
| --- | --- | --- | --- | --- | --- | --- | --- |
| **MOR** | (CuCrFeMnNi)_3_O_4_ | 110 | 1.624 | | 1 M KOH + 0.5 M MeOH | Ni form | ^[1]^ |
|  | Ni_0.75_Cu_0.25_ | 168 | 1.822 | | 1 M KOH + 0.5 M MeOH | Ti substrate | ^[2]^ |
|  | CuCoP_x_ | 287 | 1.666 | | 1 M KOH + 1 M MeOH | Glassy carbon electrode  (GCE) | ^[3]^ |
|  | Cu@CoO_x_-CLs | 468 | 1.85 | | 1 M KOH + 1 M MeOH | GCE | ^[4]^ |
|  | Cu_1_Ni_2_-S/G | 498 | 1.50 | | 1 M KOH + 1 M MeOH | Rotating disk electrode (GCE) | ^[5]^ |
|  | CuO/Co(OH)_2_ | 764 | 1.65 | | 1 M KOH + 3 M MeOH | GCE | ^[6]^ |
|  | Ce-CoCu-LDH | 877 | 1.67 | | 1 M KOH + 1 M MeOH | GCE | ^[7]^ |
|  | Commercial Pt/C | 560 | 0.78 | | 1 M KOH + 0.5 M MeOH | GCE | This work |
|  | CuCo_2/1_ | 707 | 1.50 | | 1 M KOH + 0.5 M MeOH | GCE | This work |

**Table S2** Mass activities of CuCo_2/1_ and the state of the art for EOR.

|  | **Sample** | **Mass activity (mA∙mg^-1^)** | **Potential (V vs. RHE)** | **Electrolyte** | **Supporter** | **Reference** |
| --- | --- | --- | --- | --- | --- | --- |
| **EOR** | Cu:Co 80:20 | 38 | 1.60 | 1 M KOH + 0.1 M EtOH | GCE | ^[8]^ |
|  | α-Ni(OH)_2_ | 49 | 1.55 | 1 M KOH + 1 M EtOH | GCE | ^[9]^ |
|  | Co_2_NiO_4_ | 98 | 1.60 | 1 M KOH + 0.1 M EtOH | GCE | ^[10]^ |
|  | β-Ni(OH)_2_/NiFe_2_O_4_ | 123 | 1.524 | 1 M KOH + 1 M EtOH | GCE | ^[11]^ |
|  | ZnCo-LDH-CH_3_COO^-^ | 345 | 1.666 | 1 M KOH + 1 M EtOH | GCE | ^[12]^ |
|  | NiAl-LDH-NSs | 471 | 1.50 | 1 M KOH + 1 M EtOH | GCE | ^[13]^ |
|  | Cu_1_Ni_2_-S/G | 1000 | 1.49 | 1 M KOH + 1 M EtOH | Rotating disk electrode (GCE) | ^[5]^ |
|  | Commercial Pt/C | 316 | 0.68 | 1 M KOH + 0.5 M EtOH | GCE | This work |
|  | CuCo_2/1_ | 1266 | 1.50 | 1 M KOH + 0.5 M EtOH | GCE | This work |

**Table S3** Values for the equivalent circuits of Figure 3d.

|  | **R_s_ (Ω)** | **R_ct_ (Ω)** | **CPE1-T** | **CPE1-P** |
| --- | --- | --- | --- | --- |
| **MOR** | 5.14 | 16.78 | 0.0037171 | 0.9771 |
| **EOR** | 5.48 | 11.73 | 0.0026932 | 0.9336 |

**Table S4** Comparison of Faradaic Efficiencies for MOR and EOR between CuCo_2/1_ NCs and representative nanocatalysts reported in the literature at different voltages.

| **MOR** | | | **EOR** | | |
| --- | --- | --- | --- | --- | --- |
| CuCo_2/1_ | 82% @ 1.52 V | this work | CuCo_2/1_ | 83.3% @ 1.50 V | this work |
| CuONS/CF | 86.1% @ 1.27 V  97.0% @ 1.36 V | ^[14]^ | RuO_2_-based catalysts | 71% @ 1.40 V  56% @ 1.45 V | ^[15]^ |
| NiCo/(Ni,Co)(OH)_x_/C | 48.7% @ 0.8 V  ~100% @ 1.55 V | ^[16]^ | Ni_0.75_Co_0.25_Se_2_ | 82.9% @ 1.6 V  76.6% @ 1.5 V  51.9% @ 1.4 V | ^[17]^ |
| PtRuNiCoFeGaPbW HEA | 55% @ 0.70 V  47% @ 0.80 V  25% @ 0.9 V | ^[18]^ | NiS | 81% @ 1.60 V | ^[19]^ |
| B/CuCo_2_O_4_ | 43% * | ^[20]^ | Co-S-P | 67% @ 1.05 V | ^[21]^ |
| NiS | 98% @ 1.60 V | ^[19]^ | IrO_2_ | 51%* | ^[22]^ |
|  |  |  | IrRuO_x_/Ti | 67%* |  |
|  |  |  | Pd_76_Sb_17_Bi_7_/C | 82%* | ^[23]^ |
|  |  |  | PtRh NWs/C | 43.4% @ 1.0 V | ^[24]^ |
|  |  |  | Co_2_NiO_4_ | 60% @ 1.55 V | ^[25]^ |
|  |  |  | NiOOH-CuO | 79.1% @ 1.35 V | ^[26]^ |
|  |  |  | Ni(OH)_2_ NSs | 92% @ 1.62 V  79% @ 1.52 V | ^[27]^ |

* Voltage not specified due to lack of explicit information in the cited literature.

**Table S5** Summary of potentials at different stages of the multistep chronopotentiometry in Figure 6a.

|  | | Hybrid methanol electrolysis | | Hybrid ethanol electrolysis | | | |
| --- | --- | --- | --- | --- | --- | --- | --- |
| Current density (mA∙cm^-2^) | Time (h) | Cell voltage (V) | Difference (V) | Cell voltage (V) | | | Difference (V) |
| 10 | 0 | 1.40 |  | | 1.41 |  | |
|  | 6 | 1.43 | +0.05 | | 1.43 | +0.06 | |
| 20 | 6 | 1.48 |  |  | 1.49 |  |  |
|  | 12 | 1.52 | +0.05 | | 1.52 | +0.06 | |
| 30 | 12 | 1.57 |  |  | 1.58 |  |  |
|  | 18 | 1.58 | +0.04 | | 1.59 | +0.05 | |
| 40 | 18 | 1.62 |  |  | 1.64 |  |  |
|  | 24 | 1.63 | +0.03 | | 1.63 | +0.05 | |
| 50 | 24 | 1.66 |  |  | 1.68 |  |  |
|  | 30 | 1.70 | - | | 1.69 | - | |
|  | 36 | 1.73 | -0.04 | | 1.73 | -0.03 | |
| 40 | 36 | 1.69 |  |  | 1.70 |  |  |
|  | 42 | 1.68 | -0.05 | | 1.69 | -0.07 | |
| 30 | 42 | 1.63 |  |  | 1.62 |  |  |
|  | 48 | 1.63 | -0.05 | | 1.63 | -0.08 | |
| 20 | 48 | 1.58 |  |  | 1.55 |  |  |
|  | 54 | 1.59 | -0.08 | | 1.53 | -0.10 | |
| 10 | 54 | 1.51 |  |  | 1.43 |  |  |
|  | 60 | 1.52 |  | | 1.44 |  | |
|  |  | Overall, above | +0.12 | |  | +0.03 | |
| Refresh |  |  |  | |  |  | |
| 10 | 60+0 | 1.53 |  | | 1.41 |  | |
|  | 60+10 | 1.56 |  | | 1.44 |  | |

**Table S6** Values for the equivalent circuits of Figure S12c, f.

|  |  | R_s_ (Ω) | R_ct_ (Ω) | CPE1-T | CPE1-P | R_mt_ (Ω) | CPE2-T | CPE2-P |
| --- | --- | --- | --- | --- | --- | --- | --- | --- |
| Hybrid methanol electrolysis | before | 3.59 | 0.75 | 0.0026285 | 0.63015 | 74.40 | 0.050461 | 0.76747 |
|  | after | 3.47 | 3.85 | 0.00098373 | 0.76730 | 5.37E^+15^ | 0.046196 | 0.46900 |
|  | refresh | 3.31 | 8.49 | 0.00063222 | 0.85688 | 5.40E^+9^ | 0.037530 | 0.60130 |
| Hybrid ethanol electrolysis | before | 3.47 | 1.12 | 0.00026536 | 0.7874 | 104.10 | 0.036247 | 0.78822 |
|  | after | 4.46 | 3.30 | 0.00010484 | 0.76803 | 278.30 | 0.059234 | 0.64999 |
|  | refresh | 3.95 | 2.93 | 0.0013648 | 0.77403 | 351.60 | 0.061203 | 0.63194 |

**References**

[1] B. Talluri, K. Yoo, J. Kim, *J Environ Chem Eng* **2022**, *10*.

[2] X. Cui, P. Xiao, J. Wang, M. Zhou, W. Guo, Y. Yang, Y. He, Z. Wang, Y. Yang, Y. Zhang, Z. Lin, *Angewandte Chemie* **2017**, *129*, 4559.

[3] F. Zhao, H. Yang, K. Qin, G. Cui, Q. Liu, *J Taiwan Inst Chem Eng* **2021**, *126*, 244.

[4] Y. Sun, Y. Zhou, C. Zhu, W. Tu, H. Wang, H. Huang, Y. Liu, M. Shao, J. Zhong, S. T. Lee, Z. Kang, *Appl Catal B* **2019**, *244*, 795.

[5] Z. Wang, X. Liao, M. Zhou, F. Huang, K. A. Owusu, J. Li, Z. Lin, Q. Sun, X. Hong, C. Sun, Y. Cheng, Y. Zhao, L. Mai, *Energy and Environmental Materials* **2023**, *6*.

[6] L. Chen, Z. Hua, J. Shi, M. He, *ACS Appl Mater Interfaces* **2018**, *10*, 39002.

[7] Q. Liu, H. Yang, X. Liu, K. Qin, Q. Bu, *Inorg Chem* **2022**, *61*, 7414.

[8] M. Braun, G. Behrendt, M. L. Krebs, P. Dimitri, P. Kumar, I. Sanjuán, S. Cychy, A. C. Brix, D. M. Morales, J. Hörlöck, B. Hartke, M. Muhler, W. Schuhmann, M. Behrens, C. Andronescu, *ChemElectroChem* **2022**, *9*.

[9] J. J. B. Lidasan, J. A. D. Del Rosario, J. D. Ocon, *Catalysts* **2020**, *10*, 1.

[10] S. E. Michaud, M. M. Barber, K. E. Rivera Cruz, C. C. L. McCrory, *ACS Catal* **2023**, *13*, 515.

[11] L. Chen, X. Yang, Y. Gao, Y. Tian, Y. Wang, X. Zhao, X. Lei, F. Zhang, *Int J Hydrogen Energy* **2023**, *48*, 26148.

[12] H. Yang, T. Guo, K. Qin, Q. Liu, *Journal of Physical Chemistry C* **2021**, *125*, 24867.

[13] L. Xu, Z. Wang, X. Chen, Z. Qu, F. Li, W. Yang, *Electrochim Acta* **2018**, *260*, 898.

[14] X. Wei, Y. Li, L. Chen, J. Shi, *Angewandte Chemie - International Edition* **2021**, *60*, 3148.

[15] I. Kohlhaas, N. Kurig, R. Palkovits, *ChemElectroChem* **2024**, *11*, 1.

[16] A. Pei, R. Xie, L. Zhu, F. Wu, Z. Huang, Y. Pang, Y. C. Chang, G. Chai, C. W. Pao, Q. Gao, C. Shang, G. Li, J. Ye, H. Zhu, Z. Yang, Z. Guo, *Journal of the American Chemical Society* **2025**.

[17] J. Li, X. Wang, C. Xing, L. Li, S. Mu, X. Han, R. He, Z. Liang, P. Martinez, Y. Yi, Q. Wu, H. Pan, J. Arbiol, C. Cui, Y. Zhang, A. Cabot, *Chemical Engineering Journal* **2022**, *440*, 135817.

[18] Y. Lv, P. Liu, R. Xue, Q. Guo, J. Ye, D. Gao, G. Jiang, S. Zhao, L. Xie, Y. Ren, P. Zhang, Y. Wang, Y. Qin, *Advanced Science* **2024**, *11*, 2309813.

[19] J. Li, X. Tian, X. Wang, T. Zhang, M. C. Spadaro, J. Arbiol, L. Li, Y. Zuo, A. Cabot, *Inorganic Chemistry* **2022**, *61*, 13433.

[20] N. Kumar T R, S. Kamalakannan, M. Prakash, B. Viswanathan, B. Neppolian, *ACS Applied Energy Materials* **2022**, *5*, 2104.

[21] S. Sheng, K. Ye, L. Sha, K. Zhu, Y. Gao, J. Yan, G. Wang, D. Cao, *Inorganic Chemistry Frontiers* **2020**, *7*, 4498.

[22] C. Tian, X. Y. Li, V. E. Nelson, P. Ou, D. Zhou, Y. Chen, J. Zhang, J. E. Huang, N. Wang, J. Yu, H. Liu, C. Liu, Y. Yang, T. Peng, Y. Zhao, B. H. Lee, S. Wang, E. Shirzadi, Z. Chen, R. K. Miao, D. Sinton, E. H. Sargent, *ACS Energy Letters* **2023**, *8*, 4096.

[23] Q. Wang, T. Li, S. Yan, W. Zhang, G. Lv, H. Xu, H. Li, Y. Wang, J. Liu, *Inorg Chem* **2022**, *61*, 16211.

[24] Y. Zhu, L. Bu, Q. Shao, X. Huang, *ACS Catal* **2019**, *9*, 6607.

[25] S. E. Michaud, M. M. Barber, K. E. Rivera Cruz, C. C. L. McCrory, *ACS Catal* **2023**, *13*, 515.

[26] H. Sun, L. Li, Y. Chen, H. Kim, X. Xu, D. Guan, Z. Hu, L. Zhang, Z. Shao, W. C. Jung, *Appl Catal B* **2023**, *325*.

[27] Z. Zhang, Y. Dong, C. Carlos, X. Wang, *ACS Nano* **2023**, *17*, 17180.
